# Effect of MDMA-assisted therapy on mood and anxiety symptoms in advanced-stage cancer (EMMAC): study protocol for a double-blind, randomised controlled trial

**DOI:** 10.1186/s13063-024-08174-x

**Published:** 2024-05-21

**Authors:** Chiranth Bhagavan, Paul Glue, Will Evans, Lisa Reynolds, Thivya Turner, Chris King, Bruce R. Russell, Eva Morunga, Jessica Lee Mills, Geoff Layton, David B. Menkes

**Affiliations:** 1 Department of Psychological Medicine, Division of Health Sciences, University of Otago, 464 Cumberland Street, Central Dunedin, Dunedin, 9016 New Zealand; 2 Te Whatu Ora Southern, Dunedin Public Hospital, 201 Great King Street, Dunedin, 9016 New Zealand; 3 School of Pharmacy, University of Otago, 18 Frederick Street, Dunedin North, Dunedin, 9016 New Zealand; 4 Ashburn Clinic, 496 Taieri Road, Halfway Bush, Dunedin, 9010 New Zealand; 5 Mana Health, 7 Ruskin St, Parnell, Auckland, 1052 New Zealand; 6 Department of Psychological Medicine, Faculty of Medical and Health Sciences, University of Auckland, 22-30 Park Avenue, Grafton, Auckland, 1023 New Zealand; 7 Te Whatu Ora Te Toka Tumai, Auckland City Hospital, 2 Park Road, Grafton, Auckland, 1023 New Zealand; 8 University of Auckland, 6 Symonds Street, Auckland, 1010 New Zealand; 9 Department of Psychological Medicine, Faculty of Medical and Health Sciences, University of Auckland, Waikato Clinical Campus, Peter Rothwell Academic Centre, Pembroke Street, Hamilton, 3240 New Zealand

**Keywords:** 3,4-Methylenedioxymethamphetamine (MDMA), MDMA-assisted therapy, Depression, Anxiety, Advanced-stage cancer, Terminal illness, Palliative care

## Abstract

**Background:**

Symptoms of anxiety and depression are common in patients with terminal illness and multiple challenges exist with timely and effective care in this population. Several centres have reported that one dose of the serotonergic psychedelic psilocybin, combined with therapeutic support, improves these symptoms for up to 6 months in this patient group. Drawing upon related therapeutic mechanisms, 3,4-methylenedioxymethamphetamine (MDMA)-assisted therapy may have the potential to achieve similar, positive mental health outcomes in this group. Preliminary evidence also supports the tolerability of MDMA-assisted therapy for anxiety and depression in advanced-stage cancer.

**Methods:**

Up to 32 participants with advanced-stage cancer and associated depression and anxiety will be randomised in a 1:1 ratio into one of two blinded parallel treatment arms. The intervention group will receive 120 mg (+ 60 mg optional supplemental dose) MDMA-assisted therapy. The psychoactive control group will receive 20 mg oral (+ 10 mg optional supplemental dose) methylphenidate-assisted therapy. For each medication-assisted therapy session, participants will undergo two 90-min therapeutic support sessions in the week preceding, and one 90-min support session the day after the experimental session. A battery of measures (mood, anxiety, quality of life, mystical experience, spiritual wellbeing, attitudes towards death, personality traits, holistic health and wellbeing, connectedness, demoralisation, expectations, qualitative data and safety measures) will be assessed at baseline and through to the end of the protocol. Participants will be followed up until either 12 months post-randomisation or death, whichever occurs first.

**Discussion:**

This study will examine the effect of MDMA-assisted therapy on symptoms of anxiety and depression in advanced-stage cancer. Potential therapeutic implications include establishing the safety and effectiveness of a novel treatment that may relieve mental suffering in patients with life-threatening illness.

**Trial registration:**

Trial registered on Australian New Zealand Clinical Trials Registry.

Registration number: ACTRN12619001334190p.

Date registered: 30/09/2019.

URL: https://www.anzctr.org.au/Trial/Registration/TrialReview.aspx?id=378153&showOriginal=true&isReview=true

**Supplementary Information:**

The online version contains supplementary material available at 10.1186/s13063-024-08174-x.

## Administrative information

Note: the numbers in curly brackets in this protocol refer to SPIRIT checklist item numbers. The order of the items has been modified to group similar items (see http://www.equator-network.org/reporting-guidelines/spirit-2013-statement-defining-standard-protocol-items-for-clinical-trials/).

**Table:** 

Title {1}	Effect of MDMA-assisted therapy on Mood and Anxiety symptoms in advanced-stage Cancer (EMMAC).
Trial registration {2a and 2b}.	Registry name: Australian New Zealand Clinical Trials Registry. Registration number: ACTRN12619001334190p Date registered: 30/09/2019
Protocol version {3}	16 October 2023 Version 1.10 Identifier: EMMAC_Protocol_INZL1_v1.10_16Oct2023
Funding {4}	Funding for this investigator-initiated study is being sought from the Universities of Auckland and Otago and an individual donor. None of the investigators or staff associated with this study have a proprietary or equity interest of any sort in MDMA development. The Multidisciplinary Association for Psychedelic Studies Public Benefit Corporation (MAPS PBC) has donated supplies of the study drug and technical assistance to protocol development and training of therapists. MAPS PBC has not offered or provided payments to the investigators.
Author details {5a}	University of Otago Team Professor Paul Glue, Department of Psychological Medicine, Division of Health Sciences, University of Otago, 464 Cumberland Street, Central Dunedin, Dunedin 9016, New Zealand Dr Thivya Turner, Te Whatu Ora Southern, Dunedin Public Hospital, 201 Great King Street, Dunedin 9016, New Zealand Chris King, Te Whatu Ora Southern, Dunedin Public Hospital, 201 Great King Street, Dunedin 9016, New Zealand Professor Bruce R. Russell, School of Pharmacy, University of Otago, 18 Frederick Street, Dunedin North, Dunedin 9016, New Zealand Dr Geoff Layton, Ashburn Clinic, 496 Taieri Road, Halfway Bush, Dunedin 9010, New Zealand University of Auckland Team Dr Will Evans, Medical Director, Mana Health, 7 Ruskin St, Parnell, Auckland 1052, New Zealand Dr Lisa Reynolds, Department of Psychological Medicine, Faculty of Medical and Health Sciences, University of Auckland, 22–30 Park Avenue, Grafton, Auckland, 1023, New Zealand Eva Morunga, Te Whatu Ora Te Toka Tumai, Auckland City Hospital, 2 Park Road, Grafton, Auckland 1023, New Zealand Jessica Lee Mills, University of Auckland, 6 Symonds Street, Auckland 1010, New Zealand Dr Chiranth Bhagavan, Department of Psychological Medicine, Faculty of Medical and Health Sciences, University of Auckland, 22–30 Park Avenue, Grafton, Auckland, 1023, New Zealand Associate Professor David B. Menkes, Department of Psychological Medicine, Faculty of Medical and Health Sciences, University of Auckland, Waikato Clinical Campus, Peter Rothwell Academic Centre, Pembroke Street, Hamilton 3240, New Zealand
Name and contact information for the trial sponsor {5b}	The University of Otago PO Box 56 Dunedin 9054 The University of Auckland Private Bag 92019, Victoria St West Auckland 1142
Role of sponsor {5c}	The study sponsors and funders have no involvement in study design; collection, management, analysis and interpretation of data; writing of the report; and the decision to submit the report for publication.

## Introduction

### Background and rationale {6a}

#### Background

Advanced-stage cancer is associated with a high prevalence of depression, anxiety and post-traumatic stress disorder (PTSD) [1–3]. These disorders significantly impact quality of life [4], contributing to the broad term “existential distress”, one of the most challenging problems in palliative medicine [5, 6].

Moreover, several challenges exist in providing timely and effective care for this population. These include limited access to specialist psychological and psychiatric input, issues with assessment and accurate diagnosis in complex presentations, where addressing psychological issues may be less prioritised, limited training for mental health services for managing these unique and existential issues encountered in palliative medicine, side effects and interactions in often medically complex and comorbid patients, and delayed onset of action for psychological and pharmacological interventions coupled with the limited time available at the end of life [7].

Since 2007, when the National Comprehensive Cancer Care Network identified “distress” as the sixth vital sign [8], significant progress has been made towards defining a new treatment paradigm using a novel class of pharmacological agents for pathological distress associated with life-threatening illness.

3,4-Methylenedioxymethamphetamine (MDMA), a low affinity agonist of the serotonin 2A (5-HT_2A_) receptor and a potent releaser and re-uptake inhibitor of serotonin, norepinephrine, and to a lesser extent dopamine [9], has therapeutic potential for treating psychological disorders prevalent in advanced-stage cancer. MDMA’s qualitative effects have been described as pro-social and include increased feelings of sociability, extroversion, trust, acceptance, communication and empathy. Compared with psilocybin, a potent 5-HT_2A_ agonist, MDMA is less prone to cause hallucinatory effects, fear, disorientation, nausea and ego-dissolution [10–17]. Like psilocybin, MDMA increases measures of the personality domain of openness [18, 19]. Trials of single doses of psilocybin in advanced-stage cancer have shown promising results, significantly reducing anxiety and depression, and improving quality of life in the majority of participants [20–22]. This effect profile has also been reproduced in treatment-resistant depression including in an open-label study and phase 2 study [23, 24]. A large international multi-centre randomised-controlled trial (RCT) of psilocybin in the treatment of depression is currently underway [25].

#### Neurobiological mechanisms of MDMA

The neurobiological mechanisms that underlie the purported therapeutic effects of MDMA-assisted therapy are not fully understood. Current theories include the potential role of MDMA in increasing oxytocin levels, which may strengthen bonding with family members and significant others, including clinicians, thereby strengthening therapeutic alliances [26, 27]. Functional magnetic resonance imaging (fMRI) studies suggest MDMA increases ventromedial prefrontal activity and decreases amygdala activity [28], which may have positive effects on emotional regulation, avoidance behaviours and fear [29]. Interestingly, MDMA has been found to modulate interoception (how bodily signals are interpreted and received) [30, 31], abnormalities of which have been associated with both depression and anxiety disorders [30–34]. fMRI studies of participants with dysregulated interoception have shown changes in functional circuitry that correlate with regions of interest also affected by MDMA, specifically the insula [31]. MDMA is also known to increase norepinephrine release and circulating cortisol [35], possibly leading to improved emotional engagement and decreased fear responses. These neurobiological mechanisms are relevant to pain perception and the concept of central sensitisation (nociplastic pain) [36–38] and may also help explain reports of decreased pain perception in previous uncontrolled studies of MDMA [15, 39–42]. This concordance suggests a neurobiological basis for the potential therapeutic effects of MDMA in depression and anxiety in this population.

#### Pharmacokinetics

The pharmacokinetics of oral MDMA are complex but relatively well described, showing non-linear characteristics of dose versus plasma concentration [35, 43–45]. Oral MDMA undergoes hepatic metabolism and is both a substrate and inhibitor of cytochrome P450 2D6. The half-life of MDMA is 7–8 h with peak effects occurring at 1–2 h. The main active metabolites, 3,4-methylenedioxyamphetamine (MDA), 4-hydroxy-3-methoxymethamphetamine (HMMA) and 4-hydroxy-3-methoxy-amphetamine (HMA) are renally excreted. Most pharmacokinetic studies have used recreational dose equivalents of 1 to 1.6 mg/kg. Prototypical MDMA effects occur at doses above 80 mg [46], with greater hallucinatory effects at higher doses. [NO_PRINTED_FORM].

#### The role of mystical and spiritual experience in therapeutic effect of psychedelics

Psilocybin has been found to induce intense visual and cognitive effects, with profound changes in consciousness leading to purported therapeutic mystical or spiritual experiences [11, 47]. Although MDMA differs from psilocybin in both chemical structure and pharmacology [10], it may have the potential to achieve similar positive mental health outcomes without these potentially confrontational experiences and perceived psychological risk associated with high (hallucinogenic) doses of psilocybin.

#### MDMA-assisted therapy

As mentioned, MDMA directly reduces fear-related activity in the amygdala [28]. It also stimulates cortical activity (layer 5 pyramidal cells), via 5HT_2A_ receptors, similarly to classical psychedelics [10], which have also been used in the context of end-of-life distress [21, 22]. With MDMA there is no reduction or clouding of consciousness, as often occurs with sedation in the palliative care setting. Taken together, these mechanisms may allow people to address avoided psychic contents such as regrets about their life, unfinished business, problematic relationships and past traumatic experiences. It is also thought to facilitate revision of existential defences, providing the opportunity for rediscovering or strengthening a sense of meaning and purpose in life.

#### Previous research, effects and safety

##### The evidence for MDMA-assisted therapy

The therapeutic potential of MDMA has been recognised by the Food and Drug Administration (FDA) in the United States of America, which granted “breakthrough treatment” designation to MDMA in 2017 for phase 3 trials to commence in 2018 for severe PTSD. The results of this first two double-bind, placebo-controlled, phase 3 MDMA-assisted therapy studies (*n* = 90 and *n* = 104 respectively) showed significantly reduced PTSD symptoms and functional impairment in the MDMA group compared to placebo [48, 49]. Prior to these studies, phase 2 randomised-controlled pilot studies for PTSD consistently showed that MDMA-assisted therapy improves symptoms of anxiety in participants with chronic treatment-resistant PTSD, with efficacy extending up to and in some cases beyond 12 months after treatment [46, 50–53].

Other work has included a randomised, double-blind, placebo-controlled pilot study which explored the effect of MDMA-assisted therapy for social anxiety in autism spectrum disorder (ASD) [54]. This revealed promising results with improvements in social anxiety scores being significantly greater for the MDMA group compared to the placebo group at 1 month and 6 months follow-up, and no serious adverse events—psychological or medically related. Further, multiple studies have included depression scales as secondary endpoints with generally positive outcomes [46, 50, 51].

Although MDMA-assisted therapy has not been specifically investigated in the context of advanced cancer, there has been one phase 2 randomised-controlled trial using MDMA-assisted therapy for treatment of anxiety in the setting of life-threating illness. This study showed MDMA was well-tolerated and produced a mean reduction in anxiety compared to placebo, although had a small sample (*n* = 18) and group differences failed to reach statistical significance [55].

##### Safety and tolerability

Data from a pooled analysis of six phase 2 RCTs for MDMA-assisted therapy in PTSD and the two phase 3 RCTs for MDMA-assisted therapy in PTSD showed that MDMA-assisted therapy is generally and relatively safe and well tolerated [48, 49, 53]. The most frequently reported psychiatric adverse reactions in these studies were anxiety, depressed mood, insomnia, irritability and panic attack. The most common physical adverse reactions included dizziness, fatigue, headache, jaw clenching/tight jaw, muscle tightness, lack of appetite, nausea, hyperhidrosis, feeling hot, feeling cold and transient rises in blood pressure, heart rate and temperature. Most expected reactions were rated mild or moderate, were transient, and reduced in frequency over the 7 days following an experimental session. Moreover, no changes in neurocognitive function were detected. Positive suicidal ideation and non-suicidal self-injurious behaviour were reported in some participants, although the causal relationship to the psychotherapeutic process, study-drug, or to random group differences were unable to be determined. There were few serious adverse events related to suicidal ideation in the MDMA-assisted therapy groups, and no recorded suicide attempts. Significant cardiovascular adverse events in the MDMA-assisted groups were few but included an exacerbation of ventricular extrasystole, QT prolongation, irregular heartbeats and palpitations. Additionally, there was no reported abuse of externally sourced MDMA during the study treatments.

Whilst most MDMA-assisted therapy studies have involved physically healthy participants, importantly, the phase 2 RCT in participants with life-threatening illness (in those with greater physical health difficulties) showed it was well tolerated in this population [55]. In this study, there was no serious suicidal ideation or positive suicidal behaviour, and elevations in vital signs were self-limiting. Other common adverse events were similar to PTSD studies, and most adverse events were deemed to be related to cancer progression and medical interventions.

### Objectives {7}

The objectives of the current study are to assess the effect of a single dose of methylphenidate-assisted vs MDMA-assisted therapy on depression and anxiety in participants with advanced-stage cancer. Our hypothesis is that MDMA-assisted therapy will improve depression and anxiety in participants with advanced-stage cancer at 4 weeks from baseline as measured by changes in the Montgomery Asberg Depression Scale (MADRS) (primary outcome) [56], Hamilton Anxiety Rating Scale (HAM-A) and Hospital Anxiety and Depression Score (HADS) (secondary outcomes) [57, 58].

The study will also evaluate the safety and tolerability of MDMA-assisted therapy in participants with advanced-stage cancer. This will be assessed throughout the study by reported adverse events, laboratory tests, vital signs, physician supervision, concomitant medications required, suicidality via the Columbia-Suicide Severity Rating Scale (C-SSRS) [59] and performance status via the Australian-modified Karnofsky Performance Score (AKPS) [60].

Providing MDMA-assisted therapy in the advanced-stage cancer setting is a relatively novel intervention. Therefore, additional, exploratory objectives will be analysed including measures about quality of life, mystical experiences, spirituality, attitudes towards death, personality traits, holistic health and wellbeing, connectedness, demoralisation, expectations and qualitative data. Analysis or inferential testing of these measures will be considered exploratory in nature.

### Trial design {8}

This is a double-blind, randomised study, testing the superiority of MDMA-assisted therapy over a control group, replacing MDMA with a psychoactive placebo—methylphenidate, in advanced-stage cancer and associated depression and anxiety. Up to 32 participants (16 per arm) will be enrolled in the study. After screening, all participants will undergo two preparatory therapy sessions. Participants will then be randomised in a 1:1 ratio into one of the following treatment arms:

- Methylphenidate 20 mg + 10 mg optional supplemental dose, or
- MDMA 120 mg + 60 mg optional supplemental dose

On the day following the experimental session, all participants will undergo an integration therapy session. Assessments will continue to be undertaken until the 28th day following the experimental session. No treatment arm cross-over will be undertaken.

## Methods: participants, interventions and outcomes

SPIRIT reporting guidelines have been used for protocol preparation [61].

### Study setting {9}

Data will be collected at two sites in New Zealand: Dunedin and Auckland. All study visits will occur in a comfortable, monitored clinical setting. During the experimental session, participants will have two qualified therapists with them for at least 8 h after receiving their dose. The sites for these experimental sessions will provide additional, comfortable non-clinical surroundings including temperature control, an eye-mask, a couch, blankets, headphones and music. The music played will be a standardised playlist decided by the study team, in keeping with previous studies of psychedelic-assisted therapy for novel indications, to manage the potential role of music as a confounding variable and optimise experimental control for the hypotheses being tested [62].

### Eligibility criteria {10}

#### Population

All participants will be adults aged 18 and over with a diagnosis of life-threatening or other advanced-stage cancer and associated depression and anxiety. Table 1 outlines the full inclusion and exclusion criteria.

**Table 1:** Full eligibility criteria

Inclusion criteria	Exclusion criteria
(1) Diagnosed with advanced-stage cancer (Stage 4). (2) Prognosis of at least 3 months life expectancy from the time of screening. (3) Diagnostic and Statistical Manual of Mental Disorders, 5th edition (DSM-5) diagnosis of a depressive disorder, anxiety disorder and/or adjustment disorder/stress reaction. (4) MADRS score > 15 and HAM-A score > 12. (5) Aged at least 18 years. (6) Able to swallow pills. (7) The participant agrees to have study visits recorded. (8) Must agree to inform the investigators within 48 h of any medical conditions and procedures. (9) Must consent for the investigators to communicate directly with their medical team, for example, their oncologist, general practitioner (GP) and/or palliative care physician. (10) Agree to refrain from starting any new psychiatric medication and/or therapy during the study. (11) Willing to follow restrictions and guidelines concerning consumption of food, beverages, including caffeine and nicotine the night before and just prior to sessions. (12) Agree to have transportation other than driving themselves to where they are staying on the day of the experimental session. (13) Are able and willing to be contacted via telephone as necessary. (14) If of child-bearing potential, must have a negative pregnancy test and agree to use an effective form of contraception for 10 days following the last treatment session. Effective contraception includes: intrauterine device, injected, implanted, intravaginal, or transdermal hormonal methods, abstinence, oral hormones plus barrier contraception, vasectomised sole partner, or double barrier contraception. Two forms of contraception are required with any barrier method or oral hormones. Not of childbearing potential is defined as permanent sterilisation, postmenopausal, or assigned male at birth. (15) Must provide details of a contact/support person in the event of being unreachable by study staff or in the event of severe, emergent distress or suicidality. (16) Are proficient in speaking and reading English. (17) Agree to not use any medications on the Prohibited Medications list during the study. (18) Agree to the lifestyle modifications illustrated below, comply with requirements for fasting, refrain from certain medications prior to the experimental session, not participate in any other interventional clinical trials during the duration of the study, remain at the study site after the experimental session until cleared to be transported home after, and commit to medication dosing, therapy and study procedures. (19) All participants must agree to the following lifestyle modifications at enrollment and throughout the duration of the study. Participants are eligible to enrol in the study if they: a. Are willing to commit to medication dosing, therapy sessions, follow-up sessions, completing evaluation instruments, and all necessary telephone contact b. Agree to not participate in any other interventional clinical trials during the duration of this study *Leading up to experimental sessions* c. Agree to take nothing by mouth except clear liquids after 12:00 A.M. (midnight) the night before the experimental session d. Refrain from the use of any psychoactive medication not approved by the research team from baseline through study termination. e. Agree not to use caffeine or nicotine for 2 h before and at least 6 h after the initial dose during the experimental session. f. Are willing to comply with medication requirements per protocol (refer to the section on Allowed Concomitant Medications). Medications will only be discontinued after enrollment per clinical judgement of the site physician in consultation with the prescribing physician. g. Are on a stable dose of allowable opiates (per the section on Allowed Concomitant Medications) for two weeks leading up to the experimental session as determined by the study physician. During this period and throughout the study, the participant will be allowed to take usual pain medication if needed for breakthrough pain. h. Agree that, for 1 week preceding the experimental session to refrain from: • Taking any herbal supplement (except with prior approval of the research team). • Taking any nonprescription medications (with the exception of non-steroidal anti-inflammatory medications or acetaminophen/paracetamol unless with prior approval of the research team). • Taking any prescription medications (with the exception of contraception, thyroid hormones, or other medications approved by the research team).	(1) Pregnancy or lactation (2) Body mass index (BMI) < 15 (3) Recent (less than 6 months) or current use of illicit drugs including methamphetamine, heroin and synthetic cannabis. (4) Other non-prescribed drugs will prompt exclusion at the discretion of the study physician. (5) Are unable to give adequate informed consent. (6) Is taking a medication that is exclusionary or has not stopped taking an exclusionary drug for the requisite washout period. (7) Liver function test > three times the upper limit of normal or creatinine clearance < 30 mL/min (8) Upon review of medical or psychiatric history must not have any current or past diagnosis that would be considered a risk to participation in the study, including significant cardiovascular disease (see below). (9) Any participant presenting current serious suicide risk, as determined through psychiatric interview, responses to the Columbia-Suicide Severity Rating Scale (C-SSRS), or clinical judgement of the investigator, will be excluded; however, history of suicide attempts is not in itself exclusionary. Any participant likely to require hospitalisation related to suicidal ideation and behaviour, in the judgement of the investigator, will not be enrolled. Any participant presenting with the following on the baseline C-SSRS will be excluded: • Suicidal ideation score of 4 or greater within the last month of the assessment at a frequency of once a week or more. • Suicidal ideation score of 5 within the last 6 months of the assessment. • Any suicidal behaviour, including suicide attempts or preparatory acts, within 6 months of the assessment. Participants with non-suicidal self-injurious behaviour may be included if approved by the study physician. (10) Have a history of any medical condition that could make receiving a sympathomimetic drug harmful due to increases in blood pressure and heart rate. This includes, but is not limited to, a history of myocardial infarction, cerebrovascular accident, or aneurysm. Participants with other mild, stable chronic medical problems may be enrolled if the site physician, chief investigator and study physician agree the condition would not significantly increase the risk of MDMA administration or be likely to produce significant symptoms during the study that could interfere with study participation or be confused with side effects of MDMA. Examples of stable medical conditions that could be allowed include, but are not limited to, diabetes mellitus (type 2), human immunodeficiency virus (HIV) infection, gastroesophageal reflux disease, etc. Any medical disorder judged by the investigator to significantly increase the risk of MDMA administration by any mechanism will require exclusion. (11) Have uncontrolled essential hypertension using the standard criteria of the American Heart Association (values of 140/90 mmHg or higher assessed on three separate occasions). (12) Have a history of ventricular arrhythmia at any time, other than occasional premature atrial contractions (PACs) or premature ventricular contractions (PVCs) in the absence of ischemic heart disease. (13) Have Wolff-Parkinson-White syndrome or any other accessory pathway that has not been successfully eliminated by ablation. (14) Have a history of arrhythmia, other than occasional PACs or PVCs in the absence of ischemic heart disease, within 12 months of screening. Participants with a history of atrial fibrillation, atrial tachycardia, atrial flutter or paroxysmal supraventricular tachycardia or any other arrhythmia associated with a bypass tract may be enrolled only if they have been successfully treated with ablation and have not had recurrent arrhythmia for at least one year off all antiarrhythmic drugs and confirmed by a cardiologist. (15) Have a marked baseline prolongation of QT/QTc interval, for example, repeated demonstration of a QTc interval > 450 ms in males and > 460 ms in females. For transgender or non-binary participants, QTc interval will be evaluated based on sex assigned at birth, unless the participant has been on hormonal treatment for 5 or more years. (16) Have a history of additional risk factors for torsade de pointes (for example, heart failure, hypokalemia, family history of long QT syndrome). (17) Require use of concomitant medications that prolong the QT/QTc interval during experimental sessions. (18) Have any current problem which, in the opinion of the investigator or study physician, might interfere with participation.

### Who will take informed consent? {26a}

A participant information sheet (PIS) and informed consent form will be provided to participants prior to their screening visit (see Additional File 1). Participants, relatives, guardians and, if necessary, legal representatives will be given an opportunity to discuss any details of the study with study personnel. Consent will be obtained by a member of the research team at the screening visit. The informed consent form has been approved by the Northern B Health and Disability Ethics Committee (NBHDEC) and must be signed by the participant. The investigator will ensure that the participant has been given enough information, both written and oral, about the nature, possible risks, benefits and procedures that the study will entail, in a language that the participant can understand. They will be informed that participation is purely voluntary, withdrawal at any time is possible, and that non-participation in the study will in no way affect their ongoing healthcare.

If any questions, concerns, or complaints about the study arise at any stage, the participants are invited to contact the investigators. Or, if they wish to talk to someone not involved in the study, they are invited to contact an independent health and disability advocate at the Health and Disability Commission, a Māori Research Facilitator, or the Health and Disability Ethics Committee (HDEC). Participants are also invited to discuss with their general practitioner (GP), the Cancer Society, other healthcare professional, or a lawyer for independent advice.

The dated signature of the participant must document consent and confirms consent is based on information that is understood. A copy of the consent form and PIS will be given to the participant, with the original forms retained in the records of the investigator.

### Additional consent provisions for collection and use of participant data and biological specimens {26b}

All participants will be informed of, and provide consent for, the collection and use of their data and tissue for the purposes of this study, and for any mandatory secondary uses. Additional written consent will be sought for optional secondary uses of data.

Consent for the collection and use of information may be withdrawn at any time by the participant informing the study doctor. If consent is withdrawn, study participation will end and the study team will stop collecting information from the participant. Information collected up until withdrawal from the study will continue to be used and included in the study. If a participant withdraws from this study, their treatment will continue with their usual clinical team.

At the end of the project, any personal information will be destroyed immediately except, as required by the sponsoring universities’ research policy, any raw data on which the results of the project depend which will be retained in secure storage for 10 years, after which it will be destroyed.

## Interventions

### Explanation for the choice of comparators {6b}

The primary intervention will be the standard MDMA dose of 120 mg, followed 2 h later by an optional supplemental dose of 60 mg in line with previous studies [46]. This fixed dose, rather than dosing by body weight, is based on the lack of a clear, linear dose response and behavioural effects and is in keeping with previous studies as outlined in the Multidisciplinary Association for Psychedelic Studies Public Benefit Corporation (MAPS PBC) MDMA Investigator’s Brochure [63]. The initial 120 mg dose is expected to produce all the commonly reported effects of MDMA, with the supplemental dose controlling for inter-individual differences in dose response. The supplemental dose will prolong subjective drug effects without producing physiological effects significantly greater than peak effects occurring after the initial dose.

The psychoactive placebo control will be methylphenidate 20 mg plus an optional 10 mg supplemental dose. The psychoactive effects of methylphenidate (increased alertness and euphoria) bear some resemblance to some of the effects of MDMA. Pharmacokinetically, methylphenidate is a viable active placebo to MDMA, with a similar time to peak effects (2 h), a shorter half-life (2 to 3 h), and is predominantly renally excreted [64]. Whilst longer-acting methylphenidate formulations may have a similar half-life to MDMA, these are often not used clinically first-line options in stimulant-naïve patients for tolerability reasons. Methylphenidate has also been used both as an adjunct and as monotherapy for depression in advanced-stage cancer at doses ranging from 5 to 30 mg daily; however, its antidepressant effects may be short-lived [65].

These considerations reinforce the choice of methylphenidate at doses of 20 to 30 mg in this study given its likely tolerability in this population (based on these existing uses) and its sufficient psychoactive effects to act as a suitable active placebo, whilst it is unlikely to result in enduring positive effects on anxiety and depression when administered as a single dose. This will allow for comparisons to be made with MDMA-assisted therapy and help maintain blinding for participants and study staff.

### Intervention description {11a}

Informed consent will be obtained at the screening visit before performing and testing to determine a participant’s eligibility. All participants, even if found to be ineligible, will be assigned a three-digit participant identification number. Once assigned to a participant, the participant number will not be reused. Participants will then undertake the baseline assessments scheduled for the screening visit (Visit 1; see Table 2).

**Table 2:** Study schedule

	**Study period**											
	**Enrolment and Screening**	**Preparatory Session 1**	**Preparatory Session 2**	**Experimental Session / Dosing**				**Post-allocation**				**Close-out / End of Treatment**
	**Visit 1**	**Visit 2**	**Visit 3**	**Visit 4**				**Visit 5**	**Visit 6**	**Visit 7**	**Visit 8**	**Visit 9**
**Timepoint**	**- 7 to 14**	**-3 ± 1**	**-1**	**Day 1**				**Day 2**	**Day 4 ± 1 day**	**Day 8 ± 2 days**	**Day 15 ± 2 days**	**Day 28 ± 3 days**
				**Pre dose**	**Dose**	**2 hr** **4 hr ** **6 hr**	**7 hrs**					
**Enrolment**												
Informed consent	**x**											
Assign subject identification number	**x**											
Eligibility criteria	**x**											
**Interventions**												
Therapy session		**x**	**x**					**x**				
Randomisation				**x**								
Dosing					**x**							
**Assessments**												
Height & weight	**x**											
Demographics	**x**											
Medical and surgical history	**x**											
Full physical exam	**x**											
Safety Lab tests	**x**											
Urine drug screen	**x**											
12 Lead ECG	**x**											
AKPS	**x**	**x**		**x**			**x**	**x**	**x**	**x**	**x**	**x**
MINI	**x**											
C-SSRS^a^	**x**											**x**
Concomitant medications	**x**			**x**				**x**	**x**	**x**	**x**	**x**
Vital signs	**x**			**x**		**x**	**x**	**x**				
Adverse events		x	**x**	**x**		**x**	**x**	**x**	**x**	**x**	**x**	**x**
Expectancy questionnaire				**x**								
Blinding assessment							**x**					
MADRS	**x**							**x**	**x**	**x**	**x**	**x**
HAM-A	**x**							**x**	**x**	**x**	**x**	**x**
HADS	**x**											**x**
IPOS	**x**							**x**	**x**	**x**	**x**	**x**
MEQ30							**x**					
FACIT-Sp-12	**x**						**x**					**x**
DAP-R	**x**											**x**
BFI-2	**x**											**x**
WCS	**x**							**x**			**x**	**x**
Hua Oranga	**x**							**x**			**x**	**x**
DS-II	**x**							**x**			**x**	**x**
Qualitative Interview												**x**

^a^First C-SSRS is a Lifetime assessment, the subsequent test(s) are a Since Last Visit assessment

For this study, a separate study manual has been developed based on previous established manuals for MDMA-assisted therapy as used in the previous phase 2 trial for participants with life-limiting illness and recent phase 2 trials for treatment-resistant PTSD [48, 55, 66–68]. This manual has been further adapted to provide a structured approach for pre- and post- MDMA-assisted therapy and psychoeducation, with a therapeutic approach tailored towards the treatment of depression and anxiety in the context of advanced-stage cancer and specific to the needs of participants in Aotearoa/New Zealand. All therapists providing therapy in this study will be required to have a professional qualification in either counselling, social work, psychotherapy, psychology, nursing, medicine or other suitable behavioural health modality as approved by the principal investigator, and have formal training and supervision provided by The Multidisciplinary Association for Psychedelic Studies Public Benefit Corporation (MAPS PBC) [69].

After completing the screening visit and the principal investigator has confirmed that all eligibility criteria have been met, participants will be scheduled for two therapy sessions prior to the experimental session—one in the week prior and one on the second the day before the experimental session. Safety data via adverse events reporting and performance status will be assessed during the first therapy session (Visit 2), and adverse events reporting repeated during the second therapy session (Visit 3). The MAPS PBC model for MDMA-assisted therapy will be followed: two therapists (preferably one male and one female) will be present with each participant for all pre- and post- MDMA-assisted therapy sessions and will both be present for 8 h during the experimental session.

On the day of the experimental session (Visit 4), participants will have their last caffeine or nicotine use recorded and complete their vital signs and adverse events form 2 h before their scheduled dose. Participants will be randomised in a blinded fashion to the MDMA- versus methylphenidate-assisted therapy groups. All participants will receive the first dose of either methylphenidate or MDMA, each followed 2 h later by repeat vital signs and adverse events reporting. An optional supplemental dose will be administered at 2 h if eligible. Table 3 outlines the medication and dose received in each treatment arm.

**Table 3:** Dosing regimen

Treatment arm	Initial dose	Optional supplemental dose	Cumulative dose range
MDMA-assisted therapy	120 mg	60 mg	120–180 mg
Methylphenidate-assisted therapy	20 mg	10 mg	20–30 mg

All participants will undergo 8 h of supervised medication-assisted therapy in a comfortable environment. Experimental sessions will be audio and video recorded for qualitative analysis and to assess therapist adherence to the therapy manual.

Safety data will be recorded via vital signs and adverse events reporting at 4, 6, and 7 h post-dosing, and performance status at 7 h post-dosing. In addition, at 7 h post-dosing, the efficacy of blinding will be measured for both participants and therapists, followed by measures regarding subjective drugs effects to capture the extent of any mystical and spiritual experiences experienced during the experimental session.

Post-dosing support will be given throughout the 8-h period of the experimental session, with additional support available as required for 2 h onsite and via phone contact for 24 h post-dosing. There will be a 90-min psychotherapeutic integration session the day following the experimental session (Visit 5) with an optional additional integration session at the discretion of the study therapists. Outcome measures and safety data will be repeated as per the study schedule during the integration session, and during follow-up visits on the fourth, eighth, 15th and 28th days following the experimental session (Visits 6, 7, 8 and 9 respectively).

Following this, brief follow-up interviews will be conducted with participants and/or their GP at months 1, 2, 6 and 12.

### Criteria for discontinuing or modifying allocated interventions {11b}

Only the initial dose is required to be given at the experimental session. Supplemental doses should be offered if both therapists agree and unless contraindicated. Vital signs will be measured at 2 h post-dose, prior to offering the supplemental dose. If blood pressure (BP) is less than 160 and heart rate (HR) is less than 110 and/or stabilising or trending down after reassessing at 5-min intervals and both therapists agree, then the supplemental dose will be offered to the participant as an option. No other study drug dose adjustments are permitted.

The experimental session will be discontinued if unacceptable toxicity occurs, if participants report significant problems (for example, extreme distress, suicidal thinking or severe side effects) associated with study medication, or consent is withdrawn. Participants who discontinue the study treatment will continue to have the final assessments if they consent. Participants will be followed up until death or the data cut-off date.

If participants drop out of the study early, the primary reason this should be selected from the following:

- Adverse event
- Protocol violation
- Withdrawal of consent
- Lost to follow-up
- Death

The primary reason for premature discontinuation of the study may be one of the following:

- Unexpected adverse events
- Other unexpected events
- Principal investigator request

### Strategies to improve adherence to interventions {11c}

Experimental sessions will be audio and video recorded to assess therapist adherence to the therapy manual. Adherence to the treatment manual will be randomly checked by review of the video by blinded adherence raters within the study team. The site-specific study team will monitor data in real time to ensure complete data collection for all participants, including those who discontinue treatment. Sites will be required to make and document a specific number of attempts to obtain follow-up data. To ensure that all participants regardless of group assignment are treated in a similar manner, the sites will be required to follow the protocol and treatment manual delineating the minimum length of time per visit type and describing the delivery of treatment.

### Relevant concomitant care permitted or prohibited during the trial {11d}

The principal investigator or designee will instruct the participant to notify the study team about any new medications the participant takes 28 days prior to randomisation and after the start of the study drug treatment. All medications and significant non-drug therapies (including physiotherapy and blood transfusions) administered 28 days prior to randomisation and after the start of the study drug treatment must be listed on the concomitant medications form**.** All medications will continue to be recorded through 7 days after the experimental session. Throughout the entire protocol, all medications used to treat adverse events will be collected, and all changes including discontinuations or additions to medications will be collected. The study team will also inquire about concomitant medication adherence and document all information on the concomitant medications form. Participants may return to taking psychiatric medications and discontinue contraception, as required, after the last study visit.

#### Allowed concomitant medications

The site physician may prescribe necessary and appropriate medications in accordance with New Zealand regulations during the study to treat adverse events that do not respond to other management outlined in the treatment manual. Examples include concomitant benzodiazepines for uncontrolled anxiety (for example, lorazepam at modest doses and occasional use only to avoid withdrawal effects of discontinuation during the experimental session) or sleep aids in compliance with prohibited medications list. Sublingual nitroglycerin will be available on site in case of emergency and chest pain assessed as possibly cardiac in origin, in consultation with the site physician.

Gabapentin, pregabalin and certain opiates will be allowed when prescribed for pain management. The following opiates will be allowed during the study: hydrocodone, morphine and codeine. Prior to enrollment, participants who are taking opiates not included on this list such as methadone should be cross-tapered to an allowable opiate under the care of their outside prescribing physician. Opiate medications may reduce the efficacy of MDMA and may prolong QT/QTc interval, but the allowable opiates have been selected because they have the lowest potential for QT/QTc interval prolongation. Individuals using opiates for pain management will be able to continue their long-acting and breakthrough pain regimen if they have been relatively stable for the preceding 2 weeks. This will be agreed as part of screening at the discretion of the study physician.

Prescription and nonprescription medications (psychoactive and other) and herbal supplements must be reviewed by the research team. Failure to comply with protocol requirements for concomitant medications may result in withdrawal from treatment, depending on investigator and study physician judgement.

#### Prohibited medications

To be enrolled in the study, participants must:

- Refrain from the use of any psychoactive medication not approved by the research team from baseline through study termination (with the exception of gabapentinoids or allowed opiates for pain control).
- Refrain from the use of delta-9-tetrahydrocannabinol (THC)-containing cannabis products, hypericum perforatum (St. John’s Wort), or other herbs and medicines with known serotonergic effects, from baseline to study termination.
- Refrain from taking any specified herbal supplement (except with prior approval of the research team) for 1 week preceding each experimental session.
- Refrain from taking the following medications for five half-lives of the medication preceding each experimental session:
  - ◦ Any nonprescription medications (with the exception of non-steroidal anti-inflammatory medications or acetaminophen), unless with prior approval of the research team.
  - ◦ Any prescription medications, with the exception of contraception, thyroid hormones, or other medications approved by the research team.

If the prospective participant is being treated with psychiatric medications, including antidepressants and stimulants, they will be encouraged to discuss medication tapering with their outside treating physician. The medications should then be tapered in an appropriate fashion to avoid withdrawal effects and should be discontinued at least five half-lives plus one additional week for stabilisation before the experimental session to avoid the possibility of any interaction.

If a selective serotonin reuptake inhibitor, serotonin and noradrenaline reuptake inhibitor, monoamine oxidase inhibitor or other antidepressant is used between the experimental session (Visit 4) and study termination (Visit 9), this will be noted by the study staff and the participant will continue to be followed up.

If the participant is prescribed stimulant medications for attention-deficit/hyperactivity disorder at baseline, these must be discontinued five half-lives before the experimental session not restarted for 10 days after the experimental session. They may return to using them at the same dose and frequency following this.

Medications will only be discontinued after enrollment per clinical judgement of the site physician in consultation with the prescribing physician.

### Provisions for post-trial care {30}

Participants will complete and exit the study on the 28th day post-experimental session after the final questionnaires are completed. All participants will be invited to contact the research team at any point during the following 4 weeks if there are outstanding questions or concerns about the study. Participants will be followed up until either death or 12 months post-randomisation (whichever occurs earlier). This follow-up will occur via a brief monthly phone call to check-in with the participant and no formal measures will be taken. A handover document outlining the follow-up procedure will be given to the participant and their general practitioner at study completion.

Participants are considered to be on-study until one of the following circumstances occurs:

1. Death.
2. Withdrawal of consent.
3. Loss to follow-up.
4. Breach of study agreement including development of exclusion criteria or if the study physician has new concerns about participant wellbeing.

Participants whose treatment is permanently discontinued due to an adverse event or abnormal laboratory value will be followed up at least once a week for 4 weeks. Serious adverse events, or events that have a suspect relationship to the study drug, will subsequently be followed at 4-week intervals, until resolution or stabilisation of the event, whichever comes first.

If participants are injured in this study, they would be eligible to apply for compensation from the Accident Compensation Corporation (ACC) just as they would be if they were injured in an accident at work or at home. This does not mean that any claim will automatically be accepted. Participants will have to lodge a claim with ACC, which may take some time to assess. If their claim is accepted, they will receive funding to assist in their recovery.

### Outcomes {12}

The outcomes are summarised on Table 4 and their time points outlined in Table 2.

**Table 4:** Primary, secondary and exploratory outcomes

Domain	Measure	Method
**Primary outcome**		
Depression	Montgomery Asberg Depression Scale (MADRS)	Clinician-administered
**Secondary outcomes**		
Anxiety	Hamilton Anxiety Rating Scale (HAM-A)	Clinician-administered
Anxiety and depression	Hospital Anxiety and Depression Scale (HADS)	Self-administered
**Exploratory outcomes**		
Quality of Life	International Palliative Outcomes Scale (IPOS)	Self-administered
Mystical experiences	Mystical Experience Questionnaire (MEQ30)	Self-administered
Spirituality	Functional Assessment of Chronic Illness Therapy—Spiritual Well-Being 12 Item Scale (FACIT-Sp-12)	Self-administered
Attitudes towards death	Death Attitudes Profile Revised (DAP-R)	Self-administered
Personality traits	Big Five Inventory-2 (BFI-2)	Self-administered
Holistic health and wellbeing	Hua Oranga	Self-administered
Connectedness	Watts Connectedness Scale (WCS)	Self-administered
Demoralisation	Demoralization Scale-II	Self-administered
Expectations	Expectations questionnaire	Self-administered
Qualitative experience	Qualitative Interview	Clinician-administered
**Safety**		
Psychiatric screening	Mini International Neuropsychiatric Interview (MINI)	Clinician-administered
Suicidal ideation or behaviour	Columbia Suicide Severity Rating Scale (C-SSRS)	Clinician-administered
Medical screening	Physician assessment of medical and surgical history	Clinician-administered
Performance status	Australia-modified Karnofsky Performance Status (AKPS)	Clinician-administered
Physical examination	Full body physical examination	Clinician-administered
Laboratory evaluation	Blood tests including full blood count (FBC), electrolytes, liver function tests, creatinine, thyroid function and serum pregnancy test (if applicable)	Clinician-administered
Substance use	Urine drug screen	Clinician-administered
Cardiac	12-lead electrocardiogram (ECG)	Clinician-administered
Physiology	Vital signs, including weight, temperature, blood pressure, pulse and oxygen saturations (on air)	Clinician-administered
Medication interactions	Concomitant medication form	Clinician-administered
Adverse events	Adverse events form	Clinician-administered

#### Primary outcome

##### Montgomery-Asberg Depression Rating Scale (MADRS)

The MADRS is a 10-item clinician-administered outcome that evaluates the core symptoms of depression [56]. Core symptoms covered include sadness, tension, lassitude, pessimistic thoughts and suicidal thoughts. Items are rated on a 7-point Likert scale (0 = no abnormality to 6 = severe). Item responses are summed to give a single score between 0 and 60, whereby higher scores indicate greater levels of depression. The scale is relatively quick to complete. The MADRS is a reliable measure and has good internal consistency (Cronbach’s alpha = 0.89) and good interrater reliability (intraclass correlation coefficient (ICC) = 0.86) [70, 71]. Compared with the Hamilton Depression Scale, the MADRS has been found to have greater sensitivity to treatment-related changes in depression severity [72–74]. Accordingly, the MADRS is a suitable, primary endpoint for measuring changes in depression ratings for this trial’s intervention. The MADRS will be completed at baseline and at all follow-up visits after the experimental session to monitor for trends in this depression score immediately following the dosing and therapy sessions and over time. The change in MADRS score at 4 weeks after the experimental session will serve as the timepoint for analysis of this primary outcome.

#### Secondary outcomes

##### Hamilton Anxiety Rating Scale (HAM-A)

The HAM-A is a 14-item clinician-administered outcome that measures current anxiety severity [57]. Items are rated on a 5-point Likert scale (0 = not present to 4 = very severe). A total score is summed from the items and can range from 0 to 56, where < 17 indicates mild severity, 18 to 24 indicates mild-to-moderate severity and 25–30 indicates moderate-to-severe. Two subscale scores can be derived from the HAM-A, a psychic anxiety (mental agitation and psychological distress) and a somatic anxiety (physical complaints related to anxiety). Completion takes between 10 and 15 min. Interrater reliability of the HAM-A has been shown to be consistent and the scale has demonstrated satisfactory correlation coefficients indicating sufficient sensitivity to change [75]. The HAM-A has demonstrated good reliability (Cronbach’s alpha = 0.89), and its psychic and somatic subscales have also demonstrated good internal consistency (Cronbach’s alpha = 0.83 and 0.80 respectively) [76]. The HAM-A was also a primary outcome in a similar study measuring the effects of psilocybin in cancer patients with life-threatening diagnoses and symptoms of anxiety and depression [21]. Therefore, the use of HAM-A will also allow for comparisons to be made between MDMA and psilocybin for this indication. The HAM-A will be completed at baseline and at all follow-up visits after the experimental session.

##### Hospital Anxiety and Depression Scale (HADS)

The HADS is a 14-item participant-reported outcome which measures depression and anxiety in those who are currently physically unwell [58]. Items are scored based on severity on a 4-point Likert scale from 0 to 3; however, response option wording varies across items. HADS has a recall period of 7 days. The scale consists of two subscales: the HADS-D addressing depression (seven items) and the HADS-A addressing anxiety (seven items). It takes 2 to 5 min to complete. Each subscale is scored individually, and each has a score ranging from 0 to 21. Scores less than 7 represent non cases, whilst scores of 8–10 are considered mild, 11–14 moderate and 15–21 severe. Reliability coefficients suggest good reliability (Cronbach’s alpha = 0.83) [77]. The HADS and its subscales, HADS-D and HADS-A, have also been used as primary outcome measures in another study measuring the effects of psilocybin-assisted therapy in treating clinically significant anxiety or depression in patients with life-threatening cancer [22]. This will also allow for comparisons to be made between MDMA and psilocybin for treating anxiety and depressive symptoms for this indication. Whereas the MADRS and HAM-A are clinician-rated scales, the self-reported nature of the HADS enables important subjective ratings of anxiety and depression to be assessed. The HADS will be completed at baseline and at the final follow-up visit.

#### Exploratory outcomes

##### Integrated Palliative Outcomes Scale (IPOS)

The IPOS is a 10-item scale which covers the physical, psychological and spiritual domains of life within the context of palliative care and takes about 8 min to complete [78, 79]. There are two versions of the IPOS, a clinician-completed and a participant-completed version. The latter will be used for this study. The IPOS is a valid and reliable measure, with test/re-test reliability weighted kappa values showing good agreement (range 0.50 to 0.8) [80]. The IPOS will enable the collection of useful information regarding subjective changes in functioning and quality of life following this intervention across these important domains at the end of life. The IPOS will be completed at baseline and at all follow-up visits after the experimental session.

##### Mystical Experience Questionnaire (MEQ30)

The MEQ30 is a 30-item self-report measure that evaluates a single mystical experience evoked by hallucinogens [81]. Participants are asked to consider to what degree they experienced a number of phenomena during their session on a 6-point Likert scale ranging from 0 = none, not at all, to 5 = extreme. The MEQ30 was derived from the MEQ43 and has a four-factor structure: mystical (including items from the internal unity, external unity, noetic quality and sacredness scales of the MEQ43), transcendence of time and space, positive mood and ineffability [82]. The four factors have demonstrated excellent reliability based on Cronbach’s alpha (mystical = 0.97, positive mood = 0.92, transcendence of time and space = 0.86, ineffability = 0.9) [81]. The MEQ-30 takes 5 min to complete. The MEQ30 will be completed at 7 h post-dose during the experimental session.

##### Functional Assessment of Chronic Illness Therapy—Spiritual Well-Being; The 12-item Spiritual Well-Being Scale (FACIT-Sp-12)

The FACIT-Sp-12 is a brief 12-item self-report scale designed to measure spiritual well-being in chronic or life-threatening illness [83]. It is a part of the wider FACIT measurement system that includes scales assessing health-related quality of life. Items are scored on a 5-point Likert scale (0 = not at all to 4 = very much) with a recall period of 7 days. The FACIT-Sp-12 consists of three subscales: peace, meaning and faith. Scores are calculated for each subscale by summing the item scores, multiplying by four and dividing by the number of items answered. A total score can be calculated by summing the scores of the three subscales [84]. Internal reliability of the subscales has been shown to be good (Cronbach’s alpha = 0.81 to 0.88) [83]. The FACIT-Sp-12 will be completed at baseline, 7 h post-dose during the experimental session and at the final follow-up visit.

One of the secondary aims of this study will be to explore the therapeutic overlap of MDMA and psilocybin. It has been proposed that the improvements reported in previous studies in mental health outcomes due to psychedelics such as psilocybin may depend on the mystical or spiritual experiences of participants [21, 22, 47]. As noted earlier, these experiences are less prominent with MDMA [11], and MDMA may achieve similar positive mental health outcomes without the potentially confrontational experiences and perceived psychological risk associated with high doses of psilocybin. By using validated measures for mysticism and spirituality, such as the MEQ and FACIT-Sp-12 respectively, this study will explore the possible causal role of mystical and spiritual experiences induced by psychedelic and related compounds in the treatment of anxiety and depression in cancer patients.

##### Death Attitudes Profile Revised (DAP-R)

The DAP-R is a 32-item self-report scale that measures attitude towards death. It consists of several statements related to different attitudes towards death [85]. Response categories are scored from 1 to 7 (1 = strongly disagree to 7 = strongly agree). There are five dimensions in the DAP-R, Fear of Death (7 items), Death Avoidance (5 items), Neutral Acceptance (5 items), Approach Acceptance (10 items) and Escape Acceptance (5 items). For each dimension, a mean scale score can be computed by dividing the total scale score by the number of items forming each scale. Cronbach’s alpha of the DAP-R dimensions range from 0.65 (Neutral Acceptance) to 0.97 (Approach Acceptance) and stability coefficients range from 0.61 (Death Avoidance) to 0.95 (Approach Acceptance). Overall, the scale demonstrates very good reliability and requires 5 min for completion [86]. The DAP-R will allow for exploration of the changes in these broader existential concerns faced at the end of life. The DAP-R will be completed at baseline and at the final follow-up visit.

##### Big Five Inventory-2 (BFI-2)

The BFI-2 is a 60-item scale which takes about 10 min to complete and is based on the five-factor model of personality that includes the following trait dimensions: Neuroticism, Extraversion, Openness to Experience, Agreeableness and Conscientiousness [87]. Further, this inventory operationalises a hierarchical conceptualisation of 15 specific facet traits, three within each domain, guided by previous research on hierarchical personality structure and measurement [88–92]. Items are scored on a 5-point Likert scale (1 = disagree strongly to 5 = agree strongly) with reverse option wording across some items. Scores are calculated for each domain and facet trait through item aggregation. When tested, the BFI-2 five domain scales had alpha reliabilities of 0.83 to 0.85 between samples, and retest reliabilities of 0.76, indicating high reliability. The alpha reliabilities for the 15 facet scales ranged from 0.66 to 0.85 in each sample, with retest reliabilities ranging from 0.66 to 0.83 indicating adequate to high reliability. Absolute correlations between domain scales averaged 0.20 to 0.24 between samples. For facet traits, within-domain correlations averaged 0.54 to 0.55, whereas absolute between-domain correlations averaged between 0.16 and 0.19 between samples. Taken together, these results reflect strong discrimination between the BFI-2 domains and moderate discrimination among the facets within each domain [87].

As outlined earlier, like psilocybin, MDMA has previously been shown to increase measures of the personality domain “openness” [18, 19]. The BFI-2 will allow examination of the extent and possible causal role of changes in personality dimensions, such as openness, induced by MDMA in the treatment of anxiety and depression in cancer patients and enable comparisons with psilocybin in this regard. The BFI-2 will be completed at baseline and at the final follow-up visit.

##### Hua Oranga

The Hua Oranga is based on the four components of Te Whare Tapa Whā, a Māori model of health [93–95]. These four components are as follows: Wairua—spirit, Hinengaro—mind, Tinana—body and Whānau—family. The components are connected and contribute to Māori holistic health and wellbeing, such that deficits in any one area would be viewed as unhealthy. The self-rated version (tāngata whai ora) will be completed in this study and requires 10 min to complete. The Hua Oranga will examine changes in these broader health and wellbeing outcomes in a culturally relevant manner to the Aotearoa/New Zealand context. The Hua Oranga will be completed at baseline and at 1 day, 2 weeks and 4 weeks post-experimental session.

##### Watts Connectedness Scale (WCS)

The WCS is a 19-item self-report scale that measures connectedness to self, others and the world [96]. Previous participants of studies investigating psychedelic-assisted therapy have reported renewed self-worth, a shift to feeling more connected with others and a new appreciation of the world we live in [97]. Given the relevance of these factors at the end of life, the WCS will examine any changes induced by MDMA and any causal role in the treatment of anxiety and depression in these patients. The scale has been adapted for the New Zealand context by Alesha Wells and colleagues at The University of Auckland with the addition of Māori terms and two new items that assess connection to whakapapa (ancestry) and whenua (land). The WCS will be completed at baseline and at 1 day, 2 weeks and 4 weeks post-experimental session.

##### Demoralization Scale-II (DS-II)

The DS-II is a 16-item self-report scale for measuring demoralisation. The original Demoralization Scale was a 24-item self-report measure to capture the important expression of existential distress in palliative care [98]. The refined DS-II was developed with a reduced number of items and simplified response format to facilitate a more user-friendly measure of demoralisation in the advanced care setting [99, 100]. It is based on a 2-factor model, comprising two 8-item factors: Meaning and Purpose, and Distress and Coping Ability. Each item is rated on a 3-point Likert scale, scored from 0 to 2 (0 = never, 1 = sometimes and 2 = often). Higher scores are indicative of higher levels of demoralisation. A total score is summed from the items and can range from 0 to 32. The DS-II has demonstrated good internal validity for all patients (Cronbach’s alpha = 0.89) and test–retest reliability in symptomatically stable patients (intraclass correlation = 0.80). The DS-II will therefore enable exploration of these important existential concerns faced at the end of life. The DS-II will be completed at baseline and at 1 day, 2 weeks and 4 weeks post-experimental session.

#### Expectations

It is important to investigate participant expectations as they are likely to impact experience. In addition, significant effects from the presence and impact of the research and researchers are to be expected. The placebo effect, enhanced by expectancy bias, is a major obstacle to objective outcome measurements and the scientific understanding of the causal mechanisms of any therapeutic effect [101]. To control for such factors, participant expectations will be assessed using a modified version of an expectancy questionnaire that has been previously used in the evaluation of psychedelic therapies [102]. This will be completed prior to receiving the dose on the day of the experimental session.

#### Qualitative interview

We aim to gain a richer understanding of participant’s experience of MDMA-assisted therapy and their experience of trial participation through a qualitative interview with their nominated support person (for example, a family member, spouse or close friend). This interview will be conducted by a researcher who is independent from all other aspects of trial delivery. Support has been shown to improve outcomes for all parties in cancer research [103]. MDMA has also been shown to elicit improvements in relationships that may provide additional benefit to participants. Early research on MDMA-assisted therapy targeted couples, demonstrating enhanced introspection and communication between partners [15, 16]. More recent work has built on these findings, supporting general improvements in relationships with close loved ones for those with PTSD [104–106]. This qualitative interview will comprise a semi-structured interview that assesses the experience of both the cancer patient participant and their support person, relational changes and the experience of both parties of being involved in the trial. This will be completed at the final follow-up visit.

All primary and secondary outcomes will be measured by change from baseline and between treatment groups. Regarding exploratory outcomes, the expectancy questionnaire and MEQ 30 will be measured only once per participant, both during the day of the experimental session and compared between treatment groups. All other quantitative exploratory outcomes will be measured by their change from baseline and between treatment groups.

#### Safety measures

##### Mini International Neuropsychiatric Interview (MINI)

As previously outlined, modern clinical trials of MDMA-assisted therapy have shown few, serious adverse psychological effects. However, these trials have often excluded patients with personal or family histories of other concerning serious mental health features such bipolar or psychotic disorders, thereby limiting the ability to quantify or exclude these potential risks. Therefore, careful psychiatric interview will help identify individuals at who may be vulnerable or at elevated risk of developing these adverse psychological effects. This will include utilising the MINI [107]. The MINI is a structured diagnostic interview for psychiatric disorders. It takes approximately 15 min to complete. It has shown positive results regarding validity and reliability in eliciting symptoms criteria used in making psychiatric disorder diagnoses. Interrater kappa values were above 0.79 for all diagnoses, with 70% being 0.90 or higher, indicating excellent interrater reliability. Test/retest kappa values were over 0.75 for 14 of the 23 values, and only one value below 0.45, indicating very good retest reliability. The MINI will be completed at the screening visit.

##### Columbia-suicide severity rating scale (C-SSRS)

Given the inclusion of participants with depression and anxiety, it is important to screen for and monitor suicidal risk, including how MDMA-assisted therapy may impact this. The C-SSRS is a clinician-administered measure used to identify and assess individuals at risk for suicide. It measures four constructs including suicide ideation, intensity of ideation, behaviour and lethality. There are different scale versions available including a Lifetime version and a Since Last Visit version. The scale is made up of 10 categories with a binary yes/no answer format and includes follow‐up inquiries if a yes answer is given; the measure requires about 10 min for administration. The C-SSRS intensity scale for Lifetime has a Cronbach’s alpha of 0.93 and the Since Last Visit version 0.94, suggesting excellent reliability [59]. The Lifetime version of the scale will be administered at baseline. The Since Last Visit version will be administered at the final follow-up visit and whenever indicated during the study at clinician discretion if concerns regarding suicidality arise.

##### Australian-modified Karnofsky Performance Status (AKPS)

The AKPS is an adapted version of the gold standard Karnofsky Performance Status and the Thorne-modified Karnofsky Performance Status. It is a clinician-rated measure assessing overall performance status or ability to perform activities of daily living. It is widely used in palliative care settings in Australia and New Zealand and is therefore useful to monitor in cancer patients during this trial. A single score between 10 and 100 is provided based on the clinician’s observations of the participant’s ability to perform activities of daily living, work and self-care. A score of 100 indicates normal physical abilities with no evidence of disease whilst decreasing numbers indicate reduced ability [60]. AKPS will be recorded at baseline, first therapy session, experimental session and all follow-up visits.

#### Medical evaluation

MDMA is known to transiently increase heart rate, blood pressure and temperature in a dose-dependent manner that is generally not problematic for physically healthy individuals. However, clinical research into MDMA remains nascent and additional potential medical risks, including risks to pregnancy, are not known. Moreover, this study will examine the effects of MDMA in cancer patients who are likely to experience associated physical health symptoms, underlying medical comorbidities and medications which may increase the propensity for adverse effects and interactions. It is therefore important to conduct a thorough medical screening to ensure participants at significantly elevated risk are excluded. The medical evaluation will be completed at the screening visit, as required in response to an adverse event, or as clinically indicated. This baseline evaluation will comprise:

- Physician assessment of medical and surgical history.
- A total body examination including general appearance, skin, neck, thyroid, eyes, ears, nose, throat, lungs, heart, abdomen, back, lymph nodes, extremities and basic nervous system.
- Blood tests including full blood count, electrolytes, liver function tests, creatinine, thyroid function and serum pregnancy test if applicable.
- Urine drug screen to assess for and mitigate risks associated with substance misuse or interactions.
- 12-lead electrocardiogram (ECG) to assess for any pre-existing evidence of cardiac disease or arrhythmia.
- Vital signs including weight, body temperature, blood pressure, pulse and oxygen saturations (on air). In addition to recording these at baseline, these will also be recorded multiple times during the experimental session and once on the day following the experimental session.
- Concomitant medications will be recorded at baseline, experimental session and all follow-up visits to assess for and mitigate risks associated with medication interactions, such as serotonin syndrome and QT/QTc prolongation.

#### Adverse events

All adverse events captured by the above measures or otherwise will be recorded on the adverse events form completed at each visit following screening, including multiple times during the day of the experimental session to closely detect any treatment-emergent adverse events during the acute effects of the medication.

### Participant timeline {13}

**Fig. 1:**
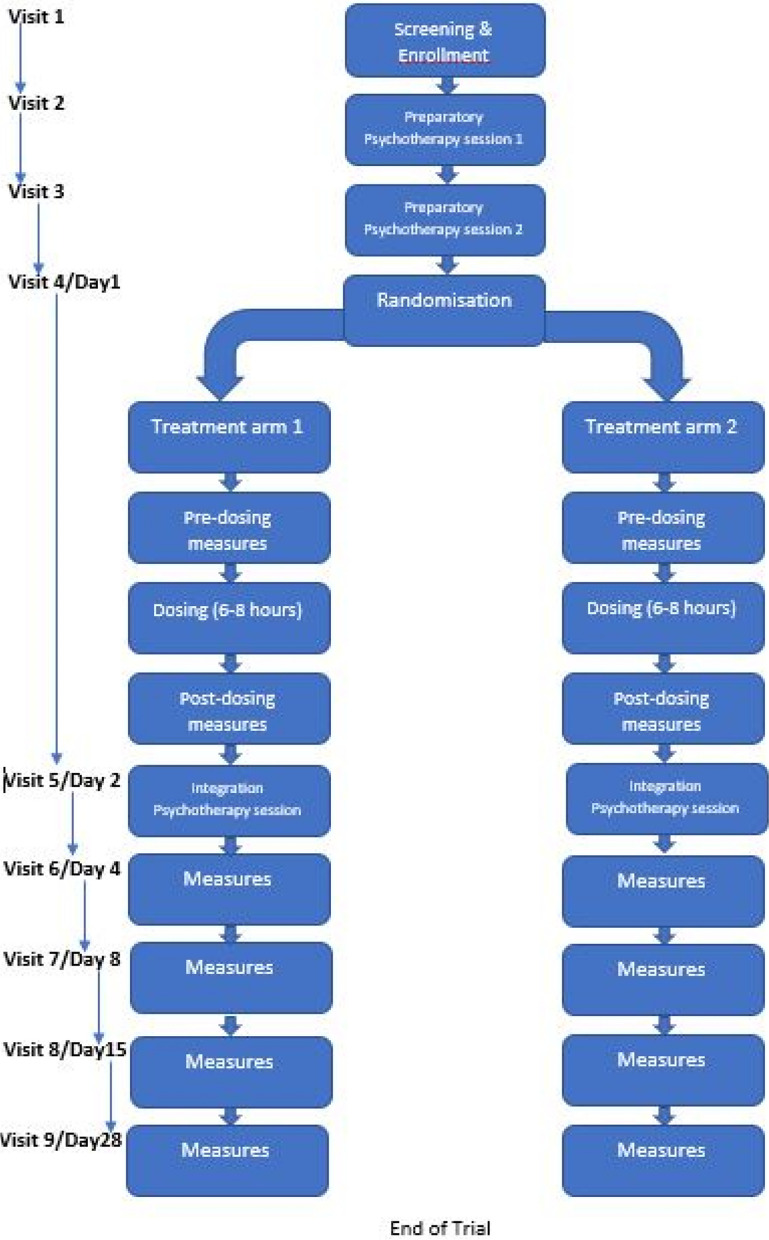
Timeline of events

Participant timeline is shown in Fig. 1.

### Sample size {14}

The phase 2 study on MDMA-assisted therapy for patients with life-threatening illness (mainly terminal cancer) and associated anxiety reported a mean difference of 14.7 (standard deviation [SD] = 14) in Spielberger Trait Anxiety Index, with 13 treated with MDMA and five treated with placebo [55]. Based on these findings and with 16 subjects in each arm, we calculate that our study has 82% power to detect a difference between treatment arms, with alpha = 0.05.

### Recruitment {15}

General practitioners, Hospice New Zealand, hospital oncology services and palliative care doctors outside these settings will be approached and provided information, the participant information sheet and a pre-screening referral form to help identify and refer patients with a diagnosis of life-threatening or other advanced-stage cancer and associated depression and anxiety. Participants will be sent study information and given the opportunity to discuss this with the research team prior to recruitment. There will be no cost to participate in this research study. If required, parking and travel costs will be reimbursed. Further strategies to promote recruitment will include a study website for referrers and participants, and a press release upon commencement of the study.

## Assignment of interventions: allocation

### Sequence generation {16a}

On the day of the experimental session, participants will be randomised in 1:1 allocation in a blinded fashion to the MDMA-assisted therapy group or the methylphenidate-assisted therapy group, stratified by clinical site and gender. A computer-generated randomisation schedule will be used to assign subjects to treatments in a 1:1 ratio.

### Concealment mechanism {16b}

Randomisation will be managed via an Interactive Web Response System (IWRS) based on a centralised randomisation schedule developed by an independent third-party vendor to maintain blinding. Enclosed treatment assignments will be serially numbered in opaque, sealed envelopes and opened sequentially after the participant’s name and other details have been written on the appropriate envelope [108].

### Implementation {16c}

The principal investigator will enrol participants once confirming that all eligibility criteria have been met. A member of the research team who is independent to any screening, data collection, outcome measurement, therapy or clinical support will assign participants to interventions.

## Assignment of interventions: blinding

### Who will be blinded {17a}

Participants, site staff and the sponsor-investigator will remain blinded to subject group assignment until after database lock at the end of the study.

To further minimise bias in measuring treatment efficacy, the sponsor-investigator will use blinded staff not directly involved in therapy to administer the primary outcome measure via in-person, live video or phone interviews. The raters are blinded to treatment assignment and will only evaluate participants at baseline and at the assessments scheduled after their experimental session. Participants will be instructed to withhold their opinion on treatment group assignment from raters. The participants, MAPS PBC and site staff will not see unblinded primary outcome measure results until after database lock at the end of the study. Changes in study management and analysis, if any, will be made by the primary investigator, site staff or study physician who are blinded to group assignment and primary outcome measure data.

The efficacy of the blinding will be measured by asking therapists and participants to rate their opinion on treatment group assignment and the confidence of their guess by using a 3-point Likert scale at the conclusion of the experimental session.

### Procedure for unblinding if needed {17b}

If there is an emergency requiring knowledge of a participant’s allocation, the blind may be broken for an individual participant through the IWRS. Upon database lock, the study will be unblinded and participants will be notified of treatment assignment.

## Data collection and management

### Plans for assessment and collection of outcomes {18a}

A registered nurse or research assistant at each site will conduct the 12-lead ECG, height, weight, vitals, urine drug screen and all baseline instruments required for eligibility during the screening visit (Visit 1). Remaining, scheduled self-administered instruments will be emailed to the participant to complete following this visit. Laboratory samples will be taken and processed by LabTests, Auckland and Southern Community Labs, Dunedin. Psychological screening via the MINI and medical screening for previous medical and surgical history, concomitant medications, physical examination and interpretation of all medical investigations will be undertaken by the study physician at each site. Concomitant medications entered into the database will be coded using the World Health Organisation Drug Reference List, which employs the Anatomical Therapeutic Chemical classification system. Coexistent disease and adverse events will be coded using the Medical Dictionary for Regulatory Activities terminology.

During the experimental session and all surrounding therapy sessions (Visits 2 to 5), the therapists will collect the scheduled outcome measures and safety data.

Scheduled outcome measures and safety data for the follow-up visits (Visits 6 to 9) will be collected by a research assistant either in-person or via audio-visual meetings. Training on how to use the instruments and forms will be provided to site staff at both the Auckland and Dunedin sites. Scheduled self-administered instruments will continue to be emailed to the participant following each visit.

The participant will be given sufficient space and time to complete the questionnaires. The study coordinator will check the questionnaire for completeness and encourage the participant to complete any missing responses. The scoring of the questionnaires will be handled as specified by the instrument developers. A description of these instruments, including reliability and validity, where known, has been provided earlier.

It is the investigator’s or study team member’s responsibility to ensure completion of the case report form (CRF) for each enrolled study participant. The staff member’s signature on the CRF, either in writing on paper-based CRFs or via electronic confirmation on the electronic CRF, serves to attest that the information contained in the CRF is true, accurate and reliable. At all times, the investigator has full responsibility for the accuracy, legibility, completeness and timeliness of all data.

### Plans to promote participant retention and complete follow-up {18b}

Participants will be supported throughout the study with the therapists and clinical support team available by telephone during business hours. In addition, there will be an on-call telephone support service from the start of the MDMA-assisted therapy session until the following morning (24 h). Participants who discontinue the study treatment will continue to have the final assessments.

A participant is considered “evaluable” and eligible for the modified intention-to-treat (*m*ITT) analysis if they have completed the experimental session and three MADRS assessments in total. A participant is considered “evaluable and completed per protocol” if they have completed the experimental sessions and all assessments as planned. These participants will be included in the *m*ITT analysis set and the per protocol analysis set. A participant is considered “evaluable and early termination” if they have completed the experimental session and three MADRS assessments in total but terminated early. These participants will be included in the *m*ITT analysis. A participant is considered to be in good standing with the clinical site if, in the opinion of the investigator and/or therapy team, the participant was compliant with protocol requirements, even if they were unable to complete all study visits.

### Data management {19}

Each participant will have a study file that contains the paper-based CRF and source documents, whilst the bulk of the data capture and the CRF will be managed via the online Research Electronic Data Capture (REDCap) software hosted at the University of Auckland [109]. REDCap is a secure, browser-based application for managing online surveys and databases. Paper-based files will include results from psychological and medical screening and investigations. These will be appended to each participant’s paper-based CRF, and key findings entered into REDCap. Relevant data from the participant eligibility criteria, demographics, height, weight, vitals, adverse events, concomitant medications, laboratory tests, blinding assessment, expectancy questionnaire, MADRS, HAM-A, HADS, IPOS, MEQ30, FACIT-Sp-12, DAP-R, BFI-2, WCS, Hua Oranga, DS-II, AKPS, MINI and C-SSRS will be entered directly into REDCap.

Audio-visual recordings of experimental sessions will be transferred and stored using encrypted, secure technology in password-protected files. These recordings will also be shared overseas to USA to MAPS PBC using secure encrypted technology platforms strictly for certification of training by specified MAPS supervisors. Due to the sensitivity of this material, all video data will be held on secure encrypted databases and transferred using secure encrypted digital platforms in line with the MAPS privacy policy. Shared video content will be password-protected, non-downloadable and time-limited. Qualitative interviews will be audio recorded via zoom to a local device, whether the interview is online or in person. These recordings will also be stored on a University server in password-protected files and deleted once the interview has been transcribed. Transcripts will be de-identified. Participants will be given the opportunity to read their transcript and request changes or withdraw their data up to 14 days after being provided with the transcript. Experimental session recordings and qualitative transcripts will be stored securely on the University server in password-protected files and kept for 10 years, after which it will be destroyed according to the University research code of conduct guidelines.

Data management will be carried out to a standard of security and confidentiality consistent with Good Clinical Practice (GCP). Data will be handled only by the research team and will be held at the Faculty of Medical and Health Sciences, University of Auckland, and the Department of Psychological Medicine, University of Otago. An original, unprocessed version of every data file will be kept on the REDCap servers which may only be modified by a University of Auckland information technology systems administrator, ensuring the fidelity and audit capability of all electronic data.

The investigator must ensure that the information required by the protocol must be entered onto the CRF. The investigator must maintain source documents for each participant in the study, consisting of all demographic information, medical and psychological screening, medical investigations and the signed informed consent form. All information on CRFs must be traceable to these source documents in the participant’s file. All paper-based CRFs and source data documents will be scanned and held on servers in password-protected files to ensure fidelity of these data and allow future audit of extracted data.

### Confidentiality {27}

All identifiable data from paper-based files and source documents will be converted to a de-identified form at the study site, at which point it is entered into REDCap. De-identified data will carry a unique trial-specific number. Only these unique study numbers will identify participants on REDCap. The investigator will retain a log linking participant code with identifiers. After the database has been declared to be complete and accurate, it will be locked, and the treatment codes will be un-blinded and made available for data analysis. The de-identified database will remain on the University of Auckland server for 10 years.

The paper-based CRFs will be stored in locked filing cabinets in locked rooms, throughout the study. On completion of the study, scanned and electronic study-specific source documents will be archived on password-protected servers, and paper-based CRFs will be archived in sealed file boxes and locked in archive cupboards. Source documents will be retained for at least 10 years then destroyed by secure shredding and deleting of documents from protected databases.

All information generated in this study must be considered confidential and must not be disclosed to any persons not directly concerned with the study except as outlined below.

Identifiable data and tissue may be accessed by the following groups:

- The investigator and designated study staff to fulfil protocol requirements.
- Local laboratory staff to process, analyse and report blood samples.
- Study monitor(s) for eligibility confirmation and source data verification purposes.
- MAPS PBC by specified MAPS supervisors for certification of training and adherence to treatment manual.
- HDEC for legal and regulatory purposes.
- Health, regulatory or government agencies for legal and regulatory purposes.
- The participant’s GP or appropriate specialist to inform them of study participation and in the event of an incidental finding of potential clinical significance.
- The Medical Office of Health in the event of a positive result for a notifiable disease.

Rarely, it may be necessary for the investigator to share identifiable data with people or groups not listed above—for example, in the event of a serious threat to public health or safety, to the life or health of the participant or another person, or if the data is required for certain legal situations.

The blood test results are required to be identifiable to ensure that in the event of major deficiencies or abnormalities, the participant’s healthcare providers will have access to relevant information. LabTests and Southern Community Labs will not be authorised to share data and/or tissue with third parties. The study team will advise participants of relevant lab results and any incidental findings.

De-identified data and tissue will be accessed and used by the following groups:

- The investigator and suitably trained and experienced study staff, to conduct the study.
- The LabTests or Southern Community Labs for sample processing, analysis and reporting purposes.
- HDEC to comply with legal and regulatory duties.
- Health, regulatory or government authorities, to comply with legal and regulatory duties.
- De-identified anonymised safety data will be sent overseas to MAPS PBC for analysis. Participants will be informed of the potential risks and cultural issues associated with sending and storing data overseas, and that there may be no New Zealand representation on overseas governance committees.

De-identified data may be included in published study results including, but not limited to, peer-reviewed publications, clinical trial registry websites, scientific meetings and regulatory or marketing submissions.

De-identified data may be made available to other researchers on request for future research as specified above and/or will be added to data from other sources to form larger datasets.

The Site Investigator has overall responsibility for data privacy and confidentiality. Any breaches of privacy would constitute a major breach of protocol and would be reported to the NBHDEC. The breach would be discussed with any affected participants and they would be given an opportunity to withdraw consent for their data to be used.

Participants have the right to access and correct their data. When participants provide informed consent, they either agree or disagree to their data to continue to be used if they withdraw or are withdrawn from the study. If they do not provide consent, all data collected up to that point in the study will be destroyed.

### Plans for collection, laboratory evaluation and storage of biological specimens for genetic or molecular analysis in this trial/future use {33}

Data will be collected from the following sources:

- Direct communication with the participant.
- Study assessments, including laboratory test results, biomedical monitoring, questionnaires, interviews and examination.
- Video recordings of MDMA-assisted therapy sessions.
- Audio recordings of qualitative interviews with the participant and elected support person.
- Participant medical records (if indicated).
- Communications with participant’s clinical care team (if indicated).

Tissue will be collected as follows:

- Urine samples will be collected from each participant on screening day. The results of the urine drug analysis will be recorded, and the urine sample will be disposed of as per policy guidelines.
- Blood samples will be collected to screen for participant’s health. Participants will be referred to LabTests or Southern Community Labs for blood testing. The laboratories are Good Laboratory Practice (GLP) compliant. The facilities are secure with tissue access restricted to those staff directly involved in their analysis. The results from these tests will be recorded and the blood collected will be disposed of as per LabTest or Southern Community Labs policy guidelines.
- Data and tissue will be collected primarily by the investigator or designated study staff. All study personnel involved in data and tissue collection will be trained in GCP, the study protocol and collection requirements.
- Data generating assessments may be performed by external third parties suitably qualified by education, training and experience.

De-identified tissue will be used for analyses as described in this protocol for screening purposes only. No tissue samples will be kept for the purposes of the study or for further analysis.

In all cases, the sponsor must be satisfied that appropriate data and tissue management plans are in place and that ethical approval for use has been obtained in accordance with local laws and regulations.

Study data and tissue analysis may lead to discoveries and inventions or development of a commercial product. The rights to these will belong to the sponsor. Participants will not receive any financial benefits or compensation from, nor have any rights to, any developments, inventions or other discoveries arising from this analysis.

Consultation regarding data and tissue management has been undertaken with the NBHDEC and local District Health Board locality approval panels including formal Māori consultation.

## Statistical methods

### Statistical methods for primary and secondary outcomes {20a}

Demographic and other baseline data (including disease characteristics) will be listed in detail. Subjective and objective data will be summarised by appropriate descriptive statistics for each treatment group.

Change in the primary outcome measure, MADRS from pre-dose to the 28th day post-dose, will be assessed with a mixed model repeated measures method, with treatment, time and the interaction of treatment with time as fixed effects, subject as random effect and baseline MADRS as covariates. The least square mean differences between MDMA and active placebo will be presented along with a 95% confidence interval. The same approach will be undertaken for the secondary outcome measures (HAM-A and HADS). A within-group meaningful change in MADRS score will be 6 points, and a between-group meaningful difference in MADRS score will be 2 points.

Data will be analysed using the Statistical Package for Social Scientists [110].

### Interim analyses {21b}

A prespecified interim analysis plan will be developed and finalised separate from this protocol. This will be conducted after *n* = 12 and will be included in the statistical analysis plan. This will include review of unblinded data by a statistician not involved with the EMMAC study, to assist in resizing the study if necessary. The principal investigator will be designated to receive written communication of recommendations following the interim review of the trial. This communication will be limited to the results needed for the decision of sample size re-estimation. No recommendations for early termination for efficacy or any other adaptations to the study design will be made based on this interim analysis. The principal investigator will make the final decision to terminate the trial.

### Methods for additional analyses (e.g. subgroup analyses) {20b}

Exploratory post hoc analyses will be completed. This will include analysing the exploratory outcomes including the IPOS, MEQ30, FACIT-Sp-12, DAP-R, BFI-2, Hua Oranga, WCS, DS-II and expectations. A detailed statistical analysis plan for these remaining outcome measures will be developed and finalised separate from this protocol.

Self-reported outcome measures and the qualitative interview provide participants and investigators with additional subjective information about the impact of a given treatment on various facets of the participant’s life. These are intended to assess symptoms, functioning, health-related quality of life, overall quality of life or combinations of these outcomes.

Qualitative analysis will be conducted for emergent themes arising via audio-visual recordings of the experimental session and content analysis from the qualitative interview regarding the experimental and therapy sessions. This will be anlaysed via thematic analysis, a systematic process for identifying patterns in qualitative data [111].

### Methods in analysis to handle protocol non-adherence and any statistical methods to handle missing data {20c}

Analyses of all efficacy endpoints will be based on the full analysis set (all evaluable randomised participants in the study) defined according to the treatment they were assigned to at randomisation in keeping with the *m*ITT principle.

Safety analysis will be performed for the safety analysis population which consists of all participants who received at least one dose of any of the study drugs with a valid post-baseline assessment. Participants will be analysed according to the treatment received.

Missing data will be managed by using multiple imputation methods.

### Plans to give access to the full protocol, participant-level data and statistical code {31c}

The corresponding author will release documentation including the full protocol, consent forms, patient information sheet and study advertisements on publication of trial results. Access to the final trial dataset will only be available to the study investigators, DMC and any other relevant regulatory bodies. Statistical code and de-identified databases will be made available upon reasonable request.

## Oversight and monitoring

### Composition of the coordinating centre and trial steering committee {5d}

The Trial Steering Committee (TSC) will comprise all authors listed and provide overall supervision of the trial. In particular, the TSC will collaboratively develop and approve the final protocol; oversee the progress of the trial, adherence to the protocol, participant safety and consideration of new information; and be responsible for publication and dissemination.

The study team has expertise in relevant disciplines, including psychiatry, palliative care, psychopharmacology, psychological therapies, pharmacy and formulation, safety assessment, setting up and running clinical trials, database creation and statistical analysis. We believe this breadth of expertise is necessary and appropriate for a safe and ethical study according to this protocol.

### Composition of the data monitoring committee, its role and reporting structure {21a}

An internal Data Monitoring Committee (DMC) will be established to review safety and efficacy. The DMC will act in an advisory capacity to the sponsor-investigator to monitor participant safety, data quality and review outcomes to ensure the trial is conducted safely and ethically and meets endpoint objectives. The DMC will be the only group reviewing group-unblinded interim data.

The composition of DMC members and its processes will be established in a separate DMC charter. DMC meetings will be held at least once every 6 months, or more frequently based on rate of recruitment, or if there are concerns about study conduct, safety, data integrity or other aspects of GCP.

### Adverse event reporting and harms {22}

Comprehensive study procedures, medical screening and eligibility criteria have been developed based on phase 2 clinical trials to exclude potential participants with pre-existing exclusionary medical conditions that would exacerbate risk.

Proper preparation and follow-up support will reduce the difficulties participants might have with acute or sub-acute psychological reactions. The potential for destabilising psychological distress will be minimised by:

- Psychiatric screening and excluding people who might be more vulnerable to it (such as people diagnosed with bipolar affective disorder type 1 or with psychotic disorders).
- Preparatory sessions of non-drug therapy before the experimental session.
- Creating an atmosphere of trust during the experimental session.
- Close monitoring.
- Phone contact with participants during the week after the experimental session.
- Integrative session(s).

The therapy teams and site physicians will be available via mobile phone throughout the study if any problem occurs when a participant is not at the site. In the event of a medical emergency or any other medical problem during an experimental session, the site physician will be immediately available by telephone, and based on assessment of the situation, they will make the decision to either evaluate the participant themselves at the site, have the therapy team call an ambulance to transport the participant to the emergency department or instruct the therapy team to take the participant to the emergency department.

Safety will be evaluated using adverse event reporting, AKPS, C-SSRS, MINI, vital signs and medical assessments and investigations. Information about all adverse events will be collected and recorded on the electronic adverse event form and followed up as appropriate.

All adverse events will be monitored by the therapy team until resolution or, if the adverse event becomes chronic, a cause can be identified. If an adverse event is unresolved when a participant terminates from the study, a clinical assessment will be made by the site physician, investigator and/or study physician as to whether continued follow-up of the adverse event is warranted.

Treatment-emergent adverse events (new or worsening from baseline) will be summarised by system organ class, incidence, frequency, severity based on common terminology criteria (CTC) grades [112], severity based on impact on normal daily activity, type of adverse event, relation to the study drug by treatment arm and whether the event qualifies as an adverse event of special interest (AESI) and/or a serious adverse event (SAE). Data from other tests (for example, ECG or vital signs) will be listed, notable values will be flagged and any other information collected will be listed as appropriate. Any statistical tests performed to explore the data will be used only to highlight any interesting comparisons that may warrant further consideration.

All SAEs will be collected from enrolment through study termination. All SAEs which occur during the trial, whether considered to be associated with study medication or not, will be reported to MAPS PBC within 24 h of the site staff’s awareness of occurrence. All SAEs will be assessed for relationship, expectedness and any required actions to address safety at the time of reporting of the event. SAEs will be evaluated by the site physician and study physician to determine if it is appropriate for the participant to continue treatment or enter follow-up.

Significant life events that may occur during the study, including disease progression, death of a loved one, loss of employment, or other hardship, may have an impact on treatment outcome. Such events will be entered as comments in REDCap, and if appropriate, described in the case study report for assessing any data outliers.

### Frequency and plans for auditing trial conduct {23}

As mentioned, study monitoring via the internally established DMC will review safety and efficacy and these meetings will occur at least once every 6 months. A monitoring report will be produced which will be circulated to all study team members, and a copy will be placed in the site file.

Medsafe and other regulatory authorities may also audit the two study sites during or after the study. This will be independent from investigators and the sponsor. The Investigator must fully cooperate with regulatory authority audits.

### Plans for communicating important protocol amendments to relevant parties (e.g. trial participants, ethical committees) {25}

Protocol amendments will be submitted to the DMC, Medsafe and the NBHDEC.

### Dissemination plans {31a}

The study team recognises the importance of communicating medical research and scientific data and their obligations to participants enrolled in a study and, therefore, encourages publication of such material in reputable scientific journals and at professional and/or academic seminars or conferences.

Results will be submitted to national and international peer-reviewed journals for publication, and presented locally, nationally and internationally at psychiatry, oncology, palliative care or other relevant conferences. A final report will be made available for wider dissemination to the public, health care professionals, policy makers and other interested groups.

It is understood by the clinical investigators that the information generated in this study may be used by MAPS PBC in connection with the development of MDMA-assisted therapy and therefore may be disclosed to government agencies in various countries. To allow for the use of information derived from the study, it is understood that the investigators may be obliged to provide MAPS PBC with de-identified study data relating to safety, demographics, dosing and outcomes, and access to all applicable study records, as needed.

## Discussion

In New Zealand, the End of Life Choice Act 2019 enables assisted dying for patients in New Zealand, and came into force on 7 November 2021 [113]. This is in keeping with the expansion of assisted dying legislation and practice internationally [114]. Whilst posing professional and ethical challenges for the medical profession, this also prompts discussion of the range of issues experienced and existing treatment options available for patients at the end-of-life [115, 116]. The compounded suffering at the end-of-life and the limitations of existing treatments for depression and anxiety in this setting provides an added impetus to develop and test new treatments.

The use of MDMA in the setting of advanced-staged cancer is a relatively new area of study that warrants further investigation. This study will be the first phase 2 clinical trial to assess the effects of MDMA-assisted therapy on mood and anxiety symptoms in advanced-stage cancer participants with an appropriate sample size, placebo controls, double-blinded design and validated measures.

Careful analysis of the efficacy of blinding and the impacts of the expectancy questionnaire will add to the understanding of the role of the placebo effect. Due to the significant perceptual effects of serotonergic psychedelics, there is significant potential for de-blinding and thereby confounding effect size estimates. Moreover, previous use of MDMA or methylphenidate may further limit the efficacy of the blind. Measurement of de-blinding will allow for adjustment of this potential confounding effect. Further, participants, therapists and all other site staff will not be unblinded until after the completion of the entire study, mitigating unblinding and nocebo effects by preventing participants and therapists to share their knowledge of whether the experiences were related to MDMA or the active placebo.

Media coverage of MDMA-assisted therapy adds to expectancy in the general population from which we will be recruiting participants. By assessing expectancy at baseline, the effects of this expectancy on outcomes will be examined. In addition, there will be a website for referrers and participants and a press release to promote recruitment. Efforts will be made to ensure balance in promoting recruitment and information sharing with neutral and unbiased information about the trial to mitigate undue or excess expectancy. In addition, referrals will only be accepted from medical professionals, not directly from participants, to further mitigate the impact of referral bias and expectancy.

The combined neurobiological and psychotherapeutic intervention in this study aligns with the current paradigm of psychedelic-assisted therapy which has so far been based on a model whereby a psychedelic substance is used as a catalyst or adjuvant of psychotherapeutic work, in the case of PTSD enabling access to and processing of repressed memories and past traumas [117, 118]. MDMA-assisted therapy was developed using the principle of “inner healing intelligence” [67, 68], whereby the participant is given non-directive psychotherapeutic support in order to find their own psychotherapeutic insight and meaning from their experience. For this study, a separate study manual has been developed based on previous established manuals for MDMA-assisted therapy and adapted specific to the needs of participants in this end-of-life setting in New Zealand.

The study will allow for assessment of the effects of this intervention on the primary and secondary outcomes of depression and anxiety symptoms as measured by the MADRS, HAM-A and HADS. The wide array of validated, exploratory outcome measures and across a breadth of additional psychological, physical, spiritual and quality of life domains will allow for assessment of the benefits which are related to and extend beyond mood and anxiety symptoms. These may include improvements in physical symptoms, including pain, nausea, fatigue and cachexia, and holistic functioning and wellbeing which are intricately linked to and exacerbated by pathological mental states in end-of-life settings. Further, these outcomes will allow for exploration of the possible causal role of changes in these factors in the treatment of anxiety and depression, such as mystical and spiritual experiences, connectedness, broader existential concerns, personality traits and expectancy. These findings may also inform the utility and design of future related studies, including for depression and anxiety in other life-threatening illnesses, and evaluating individual treatment components towards considering clinical translation and feasibility, such as comparing an MDMA-only arm vs a therapy-only arm.

Beyond testing efficacy and assessing exploratory outcomes, the comprehensive eligibility criteria, clinical screening and safety precautions will allow for considered exclusion of participants with pre-existing medical or psychiatric conditions that would exacerbate the risk of harm. The regular safety reporting and availability of therapy teams and site physicians throughout the study will allow for prompt action to address these concerns and safety analysis for better understanding the risks of this treatment.

Once participants have completed the study, they will be eligible to participate in a planned open-label extension study where they will receive MDMA-assisted therapy. This will ensure all participants are given the opportunity to receive MDMA-assisted therapy, further explore the effects of any expectancy and blinding confounds through comparisons with their first treatment intervention and allow for secondary analysis of the effects of two MDMA sessions on outcome scores in individuals receiving MDMA for a second time.

## Trial status

The EMMAC protocol is currently on version 1.10, dated 16 October, 2023. Recruiting of the trial commenced on 31 July, 2023, and is expected to run through to the anticipated completion of the trial in the third quarter of 2025. At the time of this submission, two participants have completed the study, and a further two are actively enrolled.

### Supplementary Information

Additional file 1. Participant Information Sheet and CONSENT FORM . Includes details about the study, contact details for lead researchers, study procedures, possible risks, possible benefits, and informed consent information.

Additional file 2. HDEC approval EMMAC. Ethics approval document from the Health and Disability Ethics Committee.

Additional file 3. Funding Letter – EMMAC AUOA 2021. Funding documentation letter outlining funding received from an individual donor.

## Data Availability

The corresponding author will release documentation including the full protocol, consent forms, patient information sheet and study advertisements on publication of trial results. Access to the final trial dataset will only be available to the study investigators, DMC and any other relevant regulatory bodies. Statistical code and de-identified databases will be made available upon reasonable request. Study intellectual property arising from this study will be jointly owned by the University of Auckland and University of Otago.

## Abbreviations

ACC: Accident Compensation Corporation
AESI: Adverse Event of Special Interest
AKPS: Australian Karnofsky Performance Status
BMI: Body mass index
BP: Blood pressure
C-SSRS: Columbia-Suicide Severity Rating Scale
DAP-R: Death Attitudes Profile Revised
DMC: Data Monitoring Committee
DSM-5: Diagnostic and Statistical Manual of Mental Disorders, 5th edition
ECG: Electrocardiogram
eCRF: Electronic Case Report Form
FACIT-Sp-12: Functional Assessment of Chronic Illness Therapy—Spiritual Well-Being; The 12-item Spiritual Well-Being Scale
FDA: Food and Drug Administration
fMRI: Functional magnetic resonance imaging
GCP: Good Clinical Practice
GP: General Practitioner
HADS: Hospital Anxiety and Depression Score
HAM-A: Hamilton Anxiety Scale
HDEC: Health and Disability Ethics Committee
HIV: Human immunodeficiency virus
HMMA: 4-Hydroxy-3-methoxy-methamphetamine
HMA: 4-Hydroxy-3-methoxy-amphetamine
IPOS: Integrated Palliative Outcomes Scale
IWRS: Interactive Web Response System
MADRS: Montgomery Asberg Depression Rating Scale
MAPS PBC: Multidisciplinary Association for Psychedelic Studies Public Benefit Corporation
MDMA: 3,4-Methylenedioxymethamphetamine
MEQ30: Mystical Experiences Questionnaire 30
MINI: Mini International Neuropsychiatric Interview
*m*ITT: Intent-to-treat
NBHDEC: Northern B Health and Disability Ethics Committee
PTSD: Posttraumatic stress disorder
SAE: Serious adverse event
TSC: Trial Steering Committee
