# Supplementary material for: Effect of MDMA-assisted therapy on mood and anxiety symptoms in advanced-stage cancer (EMMAC): study protocol for a double-blind, randomised controlled trial
Source: Trials. 2024 May 21;25:336. doi: 10.1186/s13063-024-08174-x (PMC11110200; doi:10.1186/s13063-024-08174-x)
Supplement: Supplementary file 1 — Additional file 1. Participant Information Sheet and CONSENT FORM . Includes details about the study, contact details for lead researchers, study procedures, possible risks, possible benefits, and informed consent information. [file 13063_2024_8174_MOESM1_ESM.pdf]

Participant Information Sheet

MDMA-assisted therapy in advanced-stage cancer (EMMAC study)

**Sponsors:**	The University of Otago<br>PO Box 56<br>Dunedin 9054	The University of Auckland<br>Private Bag 92019, Victoria St West<br>Auckland 1142
**Lead Researcher:**	Professor Paul Glue	Dr William James Evans
**Contact phone number:**	021 243 3372	021 848 788
**Ethics committee ref.:**	21/NTB/105	

You are invited to take part in a study to test medication-assisted therapy for mood and anxiety symptoms in advanced-stage cancer. Whether or not you take part is your choice. If you don't want to take part, you don't have to give a reason. If you do want to take part now, but change your mind later, you can pull out of the study at any time.

This Participant Information Sheet will help you decide if you'd like to take part. It sets out why we are doing the study, what your participation would involve, what the benefits and risks to you might be, and what would happen after the study ends. We will go through this information with you and answer any questions you may have. You do not have to decide today whether or not to participate in this study. Before you decide you may want to talk about the study with other people, such as family, whānau, friends, or healthcare providers. Feel free to do this.

If you agree to take part in this study, you will be asked to sign the Consent Form located on the last page of this document. You will be given a copy of both the Participant Information Sheet and the Consent Form to keep.

This document is 14 pages long, including the Consent Form. Please make sure you have read and understood all the pages.

WHAT IS THE PURPOSE OF THE STUDY?

Advanced-stage cancer is associated with high rates of anxiety and depression. These disorders can significantly impact quality of life. Early research suggests that MDMA (3,4-Methylenedioxymethamphetamine; ecstasy) administered alongside supportive talk therapy may be effective in reducing mood and anxiety symptoms in people with advanced-stage cancer. MDMA is an experimental drug which has not been approved for medical use except within research studies. In the USA, there have been recent studies using MDMA-assisted therapy for PTSD and anxiety in life-threatening illnesses. In New Zealand, research has been conducted on MDMA for tinnitus with no adverse effects. The purpose of this study is to investigate the safety, tolerability, and effectiveness of MDMA compared with a control

medication (methylphenidate) alongside talk therapy for participants with advanced-stage cancer who have depressive and/or anxiety symptoms. The study may result in a new treatment for anxiety and depression in advanced stage-cancer.

HOW IS THE STUDY DESIGNED?

This is a dual site double-blinded randomised controlled trial to assess the effects of MDMA-assisted therapy versus a psychoactive control treatment (methylphenidate) on depression and anxiety symptoms in patients with advanced-stage cancer.

In this study, 32 participants will be randomized on a 1:1 ratio to receive one of two oral medications (an active control or MDMA) alongside talk therapy. Participants will be monitored to assess their physical health and will complete questionnaires that assess their psychological well-being prior to treatment and post-treatment. All participants will undergo two 90-minute preparation talk sessions before undergoing a medication treatment session lasting approximately 8 hours where they will receive one of the two oral medications. The medication treatment session will be administered in a comfortable clinical setting and participants will be closely observed and supported by a qualified healthcare provider (physician, psychologist, nurse). Therapists and support staff will be available by telephone for 24 hours following dosing with MDMA. The following day, all participants will undergo a 90-minute talk therapy session to discuss their experience.

WHO CAN TAKE PART IN THE STUDY?

You may be eligible to participate in this study if you have advanced-stage cancer and have depression and/or anxiety. You will need to meet all the following criteria to take part (please refer to study protocol for more detail):

1. Diagnosed with advanced-stage cancer (stage 4) and have a prognosis of at least 3 months life expectancy from time of screening.
2. Diagnosed with a depressive disorder, an anxiety disorder, and/or an adjustment disorder/stress reaction.
3. At least 18 years old.
4. Able to swallow pills.
5. Are willing for the investigators to communicate directly with your medical team (oncologist, GP, palliative care physician, etc.) to determine study suitability.
6. Agree to refrain from starting any new psychotropic medication and/or therapy during the study period.
7. Willing to follow restrictions and guidelines concerning consumption of food, beverages, including caffeine and nicotine, the night before and the morning prior to medication dosing.
8. Can arrange transportation other than driving yourself to where you are staying on the day of medication dosing.
9. Able and willing to be contacted via telephone for all necessary telephone contacts.
10. If you are able to become pregnant, you must agree to a pregnancy test and agree to use an effective form of contraception for 10 days following the last experimental session if of child-bearing potential.
11. Can provide details of a contact/support person in the event of being unreachable by study staff or in the event of severe emergent distress or suicidality.
12. Are proficient in speaking and reading English.

You cannot take part in this study if you meet one or more of the following exclusion criteria:

1. You are pregnant or lactating.
2. Have a body Mass Index < 15.
3. Have a recent (past 12 months) or current use of illicit drugs including methamphetamine, heroin, and synthetic cannabinoids. Other non-prescribed drugs will prompt exclusion at the discretion of the study physician.
4. You are unable to give adequate informed consent.
5. Take a medication that is exclusionary or have stopped taking an exclusionary drug for less than the requisite washout period.
6. Liver function test >3 times the upper limit of normal or creatinine clearance <30 mL/min.
7. Past diagnosis that would be considered a risk for participation in the study.
8. Current serious suicide risk, as determined through a psychiatric interview, responses to C-SSRS, and clinical judgement of the investigator.
9. History of any medical condition that could make receiving a sympathomimetic drug harmful because of increases in blood pressure and heart rate. This includes, but is not limited to, a history of myocardial infarction, cerebrovascular accident, or aneurysm.
10. Have uncontrolled essential hypertension using the standard criteria of the American Heart Association.
11. Have a history of ventricular arrhythmia at any time, other than occasional premature atrial contractions (PACs) or premature ventricular contractions (PVCs) in the absence of ischemic heart disease.
12. Have Wolff-Parkinson-White syndrome or any other accessory pathway that has not been successfully eliminated by ablation.
13. Have a history of arrhythmia, other than occasional PACs or PVCs in the absence of ischemic heart disease, within 12 months of screening.
14. Have a marked Baseline prolongation of QT/QTc interval and/or require use of concomitant medications that prolong the QT/QTc interval during Experimental Sessions.
15. Have any current problem which, in the opinion of the investigator or Study Physician, might interfere with participation.
16. You are participating in any other interventional clinical trials during the duration of the study.

WHAT WILL MY PARTICIPATION IN THE STUDY INVOLVE?

If you consent to take part in this study, you be asked to attend the research clinic for a 2-hour pre-screening visit to assess your eligibility to participate. If you are eligible for the study, your participation will involve approximately 22 hours over a 6-week period and you will visit the research clinic in either Dunedin or Auckland a total of 9 times (see Table 1). Our research clinic in Dunedin is located at the Clinical Trials Unit, Cumberland Street, and in Auckland at the Clinical Research Centre, University of Auckland, Grafton.

Your participation means that you will need to agree to not use any medications on the prohibited medications list during the study. Certain opiates (hydrocodone, morphine, and codeine) are allowable when prescribed for pain management. Participants taking other

opiates than these will be asked to consult their usual physician for cross-tapering to an allowable opiate.

You will also need to undertake some lifestyle modifications in preparation for your treatment session including:

One week prior to the treatment session you will not take:

- any herbal supplement (except with prior approval from the research team),
- any nonprescription medications (with the exception of non-steroidal anti-inflammatory medications or acetaminophen/paracetamol unless with prior approval of the research team),
- any prescription medications (with the exception of birth control, thyroid hormones, or other medications approved by the research team).

Just prior / during your treatment you will need to:

- do a 12 hour fast where you will only consume alcohol-free liquid after midnight (and regular pain medications),
- have no caffeine or nicotine 2 hours before, and at least 6 hours after, your initial dosing,
- drink no more than 3 litres of electrolyte-containing liquid for the first 8 hours following dosing.

You have the option of having whānau or another support person with you at all visits. A description of study visits is outlined below.

Visit 1 (pre-screening – approximately 2 hours):
The first visit is for pre-trial screening to assess whether you are eligible to participate in this study. During the screening process we will explain to you the study and procedures. As part of being accepted into the study we will ask you to give your consent that we can inform your medical team about your participation in the study.

During this visit, a medical practitioner will ask you about your medical and psychiatric history including current and previously taken medications. The medical practitioner will also discuss with you whether there is any possibility for drug interactions between your current medications and the study drugs. You will be asked to complete a questionnaire that will assess your psychological well-being and the following health checks will be undertaken:

- A physical examination consisting of a review of body systems, and assessment of height and weight.
- Vital signs including blood pressure, respiratory rate, temperature, and heart rate measurements will be recorded.
- Blood samples will be taken to assess your kidney and liver function.
- An ECG will be done to check your heart function.
- A urine sample will be collected to test for drugs. The test is for recreational drugs and some prescription drugs. Results of the drug test will not be provided to anyone without your written consent.
- Women will provide a urine sample for pregnancy testing.

We acknowledge that for Māori, blood is a taonga, and we are asking you to provide samples of blood for safety labs. We are not storing the blood or sending it overseas. The blood

samples will be disposed of by the lab using their accredited disposal procedures. This is something you may wish to discuss with whānau.

If any of your test results are abnormal or significantly outside of the normal range, you may be asked to repeat them. The results of these screening assessments may indicate that you are not able to take part in this study. If this happens, your results will be discussed with you and suggestions for treatment provided if applicable. You may also be referred to your GP for further follow-up. If this screening process determines that you are eligible to take part in this study, you will complete the remainder of the study visits.

Visit 2 (1st of 2 preparation sessions – 90 minutes):
This session will be 3-4 days before your treatment day and will be conducted with the same therapists who will be guiding you through your treatment session. During this session, you will be asked to talk about your cancer diagnosis and how it has impacted you. The therapists will provide further information regarding the medication-assisted therapy process and answer any questions you have. For example, the therapists will discuss how to support you during treatment if you seem distressed. This could include a light touch on your shoulder or holding your hand if this is something you wanted and were comfortable with. No medication will be given at this visit.

Visit 3 (2nd of 2 preparation sessions – 90 minutes):
This talk therapy session will be conducted the day before your treatment and will continue to prepare you for your treatment. Your treatment therapists will provide you a further opportunity to discuss how the treatment day will happen and ask any remaining questions.

Visit 4 (Treatment session – approximately 8 hours):
You will attend the research clinic at a pre-arranged time. We will arrange a taxi for you if needed. Your visit to the clinic on this day will consist of the following:

- A health practitioner recording your vital signs (blood pressure, temperature, heart rate, respiratory rate).
- You completing a questionnaire about your psychological well-being.
- You being given capsules that contain either MDMA or methylphenidate depending on the group you have been randomised to. Neither you, the person administering the dose, or the therapists will know which drug you receive. Two hours later, if there are no contraindications, you will be given the option of taking a supplemental dose of the medication.
- Two therapists will be with you for the duration of the medication-assisted therapy session. This will last for approximately 6 hours. You do not have to talk for this entire period.
- This session will be video-recorded and any material will be stored securely and de-identified where possible. The footage will be used to check that our therapists are following our standardised treatment approach and will only be shown to qualified supervisors and therapists relevant to the study. You will be able to request to see this footage at any time. We will always ask your permission before showing any footage for training or educational purposes outside of the study.
- Your vital signs will be checked every two hours and you will be monitored for any adverse effects.
- Approximately 7 hours after your dose we will ask you to repeat the questionnaire and we will re-check your vital signs.

- We will complete a safety discharge checklist to ensure you are okay to go home. You will need to remain at the study site after your treatment session until cleared to be transported home afterwards.
- Your therapist team and clinical support team will be available by telephone overnight if there are any questions or concerns from you or your support person.

Visit 5 (Integration session – 90 minutes):
The day after your treatment, you will return to the research clinic for a talk therapy session with your therapists. In this session, you will discuss your experience and the therapists will support you in making sense of what happened and integrating your experience with your broader life. Your physical health will also be checked.

Visits 6-9 (Follow-up on days 3, 7, 14 and 27 post-treatment – 1 hour each):
On each of these days you will return to the research clinic so the study team can assess your physical health and psychological well-being. You will also complete a questionnaire to assess your psychological well-being.

Follow-up phone calls:
After the final visit (visit 9), our study team will contact you and/or your GP by telephone for a brief (30 minute) chat and questionnaire assessment once per month at 1, 2, 6 and 12 months after the end of the study to see how you are doing. At the one month follow-up, you and your support person will be offered the option of completing a 30-60 minute interview together with an independent researcher who was not part of your therapy team to discuss your experience of the treatment and being part of the trial.

WHAT ARE THE POSSIBLE RISKS OF THIS STUDY?

Risks from taking the study drug:
The most common side effects with oral MDMA include nausea, teeth-clenching, muscle cramps, blurred vision, chills and excessive sweating. Oral methylphenidate side effects may include feeling anxious, having poor sleep, headaches and dizziness. Any side-effects you might experience should be of mild to moderate intensity, of temporary duration, and should have resolved by the time you leave the clinic. If you would like further information on the side effects of MDMA or methylphenidate please ask the study doctor.

Risks from blood draws:
There is a slight chance of bleeding or bruising at the location where the needle is inserted for venipuncture for screening blood tests.

Reproductive Risks for Women of Child-Bearing Potential:
If you are a sexually active woman of child-bearing potential, it is very important that you do not become pregnant whilst taking MDMA or methylphenidate during this study. A woman of child-bearing potential is any pre-menopausal woman who may become pregnant. If you are unsure if this applies to you, please check with the study doctor before you start the study treatment. The risk whether MDMA may cause birth defects and/or fetal deaths is unknown and so, if you are a woman of child-bearing potential you **must use an approved form of birth control for at least a week prior to treatment. Please discuss this with the study doctor or your GP if you have any questions or concerns.**

If you do become pregnant during the study, you must tell the study doctor as soon as possible.

Reproductive Risks for Sexually Active Men:
If you are a sexually active man and have any partner who is of child-bearing potential (meaning a woman who may become pregnant) **it is very important that you use birth control** (contraception) whilst taking MDMA or methylphenidate in this study.
The effects of the MDMA on male reproduction are unknown, but it is possible that it may cause birth defects and/or fetal deaths if passed on through semen. **You and your partner must therefore use both an approved method of birth control and a barrier method of birth control** from your first dose of study drug through until 1 week after your last dose. Please discuss this with the study doctor or your GP if you have any questions or concerns.

You are responsible for informing your partner of the possible risks to an unborn child of the medicine you will be taking in this study.

If a pregnancy occurs, you must report this to the study doctor as soon as possible. Your partner will be asked to give consent for her information and her infant's information to be collected for monitoring purposes.

You must also agree not to donate sperm from the first day of the study through to 90 days after the last dose of the study drug.

WHAT ARE THE POSSIBLE BENEFITS OF THIS STUDY?

It is possible there may be no specific benefits for you after the medication-assisted therapy. It is possible that you will experience a reduction in distress and depressive/anxiety symptoms. You will have a medical examination and blood test to assess your health and you can request a copy of your medical results at any time. The results of this study could potentially help with the treatment of depressive and anxiety symptoms in people with advanced-stage cancer in the future.

WILL ANY COSTS BE REIMBURSED?

It will not cost you anything to be a participant in this study. All travel expenses related to the study will be fully reimbursed.

WHAT IF SOMETHING GOES WRONG?

If you were injured in this study, you would be eligible to apply for compensation from ACC just as you would be if you were injured in an accident at work or at home. This does not mean that your claim will automatically be accepted. You will have to lodge a claim with ACC, which may take some time to assess. If your claim is accepted, you will receive funding to assist in your recovery. If you have private health or life insurance, you may wish to check with your insurer that taking part in this study won't affect your cover.

WHAT WILL HAPPEN TO MY INFORMATION?

During this study, the study doctors/researchers, nurses and therapists will record information about you and your study participation. This includes the results of any study assessments and video footage of the MDMA-assisted therapy session. If needed, information from your hospital records and your GP may also be collected. You cannot take part in this study if you do not consent to the collection of this information.

Identifiable Information:
Identifiable information is any data that could identify you (e.g. your name, date of birth, address or audio/video footage). The following groups may have access to your identifiable information:

1. The University of Auckland and/or University of Otago research team (to complete study assessments).
2. Laboratory staff at LabTests of Southern Community Labs (to process and report your screening and safety tests).
3. The University of Auckland and/or University of Otago study monitors (to make sure the study is being run properly and that the data collected is accurate).
4. The University of Auckland and/or University of Otago and its representatives (if you make a compensation claim for study-related injury identifiable information is required in order to assess your claim).
5. The University of Auckland or University of Otago ethics committees, or government agencies from New Zealand or overseas (if the study or site is audited). Audits are done to make sure that participants are protected, the study is run properly, and the data collected is correct.
6. Your usual doctor (if a study test gives an unexpected result that could be important for your health). This allows appropriate follow-up to be arranged.
7. Specific supervisors/therapists from the Multidisciplinary Association for Psychedelic Studies (MAPS) will be shown your video footage to assess the quality of the therapy provided by the therapists.
8. Rarely, it may be necessary for the Study Doctor to share your information with other people – for example, if there is a serious threat to public health or safety, or to the life or health of you or another person OR if the information is required in certain legal situations.

De-identified (Coded) Information:
To make sure your personal information is kept confidential, information that identifies you will not be included in any report generated by the researchers or any study information sent to the University of Auckland or University of Otago. Instead, you will be identified by a code. The researcher will keep a list linking your code with your name, so that you can be identified by your coded data if needed. This list will be stored separately to your other data.

The following groups may have access to your coded information [which may be sent and stored overseas]:

- The University of Auckland and/or University of Otago, for the purposes of this study.
- People and companies working with or for the University of Auckland or University of Otago, for the purposes of this study (this may include up to 5 people in 2 companies).
- Regulatory or other governmental agencies worldwide.

The results of the study may be published or presented, but not in a form that would reasonably be expected to identify you.

Anonymised Information:
The University of Auckland or University of Otago may remove the code from your de-identified information – this is called 'anonymisation'. This makes it very difficult (but not impossible) to identify the information that belongs to you. The University of Auckland or University of Otago may share this anonymised information with The Multidisciplinary Association of Psychedelic Studies (MAPS).

Future Research Using Your Information:
If you agree, your coded information may be used for future research related to depression and anxiety in Palliative care. If you agree, your coded information may also be used for other medical and/or scientific research that is unrelated to the current study. This future research may be conducted overseas. You will not be told when future research is undertaken using your information. Your information may be shared widely with other researchers or companies. Your information may also be added to information from other studies, to form much larger sets of data.
You will not get reports or other information about any research that is done using your information. Your information may be used indefinitely for future research unless you withdraw your consent. However, it may be extremely difficult or impossible to access your information, or withdraw consent for its use, once your information has been shared for future research.

Security and Storage of Your Information:
Your identifiable information will be held securely at the University of Auckland or University of Otago during the study. After the study it is transferred to a secure archiving site and stored for at least 10 years, then destroyed. Your coded information will be entered into electronic case report forms and sent through a secure server to the University of Auckland or University of Otago. Coded study information will be kept by the University of Auckland or University of Otago in secure, cloud-based storage indefinitely. All storage will comply with local and/or international data security guidelines.

Risks:
Although efforts will be made to protect your privacy, absolute confidentiality of your information cannot be guaranteed. Even with coded and anonymised information, there is no guarantee that you cannot be identified. The risk of people accessing and misusing your information (e.g. making it harder for you to get or keep a job or health insurance) is currently very small, but may increase in the future as people find new ways of tracing information.

Your anonymised information related to safety will be sent overseas. Other countries may have lower levels of data protection than New Zealand. There may be no New Zealand representation on overseas organisations which make decisions about the use of your information. There is a risk that overseas researchers may work with information in a way that is not culturally appropriate for New Zealanders.

This research includes basic information such as your ethnic group, geographic region, age range, and sex. It is possible that this research could one day help people in the same groups as you. However, it is also possible that research findings could be used inappropriately to support negative stereotypes, stigmatize, or discriminate against members of the same groups as you.

Rights to Access Your Information:
You have the right to request access to your information held by the research team. You also have the right to request that any information you disagree with is corrected. Please ask if you would like to access the results of your screening and safety tests during the study. You may access other study-specific information before the study is over, but this could result in you being withdrawn from the study to protect the study's scientific integrity. If you have any questions about the collection and use of information about you, you should ask the researchers, therapists or study doctor.

Ownership Rights:
Information from this study may lead to discoveries and inventions or the development of a commercial product. The rights to these will belong to The University of Auckland and/or University of Otago. You and your family will not receive any financial benefits or compensation, nor have any rights in any developments, inventions, or other discoveries that might come from this information.

WHAT HAPPENS AFTER THE STUDY OR IF I CHANGE MY MIND?

You may withdraw your consent for the collection and use of your information at any time, by informing your Study Doctor. If you withdraw your consent, your study participation will end, and the study team will stop collecting information from you. Information collected up until your withdrawal from the study will continue to be used and included in the study. This is to protect the quality of the study. If you withdraw from this study your treatment will continue with your usual medical team.

At the end of the project any personal information will be destroyed immediately except that, as required by the University's research policy, any raw data on which the results of the project depend will be retained in secure storage for ten years, after which it will be destroyed.

CAN I FIND OUT THE RESULTS OF THE STUDY?

It can take quite a long time for us to analyse data from these kinds of studies. We hope to be able to share with you the final results one to two years after completion of the study. We plan to publish the results in scientific journals. You can choose to receive a summary of the results by checking the box on the Consent Form.

When you start the study, you will have a unique study number that we will use to identify you in the study documentation – to help us maintain the privacy of your information. If you wanted access to your own data later, you will need to record your identification number.

WHO IS FUNDING THE STUDY?

The study is being paid for through grants, donations and with department research funds. MDMA is being donated by the Multidisciplinary Association for Psychedelic Studies (MAPS)

WHO HAS APPROVED THE STUDY?

This study has been approved by an independent group of people called a Health and Disability Ethics Committee (HDEC), who check that studies meet established ethical standards. The Northern B Health and Disability Ethics Committee has approved this study. The scientific aspects of this study have been approved by the Standing Committee on Therapeutic Trials (SCOTT), which is part of Medsafe.

WHO DO I CONTACT FOR MORE INFORMATION OR IF I HAVE CONCERNS?

If you have any questions, concerns or complaints about the study at any stage, you can contact:

- Professor Paul Glue, Dept of Psychological Medicine; email: paul.glue@otago.ac.nz, or
- Dr. Will Evans, Mana Health; email: info@manahealth.co.nz

If you want to talk to someone who isn't involved with the study, you can contact an independent health and disability advocate on:

Phone:	0800 555 050
Fax:	0800 2 SUPPORT (0800 2787 7678)
Email:	advocacy@hdc.org.nz

For Māori health support please contact:

Mark Brunton, Kaitakawaenga Rangahau Māori (Facilitator Research Māori)

Phone	03 479 8738
Email	mark.brunton@otago.ac.nz

or

Mero Cooper, Kaiatawhai services ACH & SSH

Phone	09 307 4949 ext 29089

You can also contact the health and disability ethics committee (HDEC) that approved this study on:

Phone:	0800 4 ETHICS
Email:	hdecs@moh.govt.nz

You will be supported throughout the study with your therapists and clinical support team available by telephone during business hours. In addition, we will provide an on-call telephone support service from the start of the MDMA-assisted therapy session until the

following morning (24 hours). If at any point you are in need of additional support you can reach out to the following services:

Talk to your GP or health care professional – please ask for help from your GP or cancer care clinician. They may not realise you need some additional support. They will be able to refer you to the most appropriate support service for your needs.

Cancer Society – for cancer related support for you or your family - Monday to Friday, 8am–5pm Call 0800 CANCER (0800 226 237) or go to: https://cancernz.org.nz/

Healthline – staffed by experienced registered nurses who can provide you with health information and advice on care. Call 0800 611 116 or go to: https://www.health.govt.nz/your-health/services-and-support/health-careservices/healthline

Lifeline – for free, confidential support for any distressing situation – 24 hours a day, 7 days a week. Call 0800 LIFELINE (0800 543 354) or text HELP (4357) or go to https://www.lifeline.org.nz/

Family Services Directory – a directory of service providers that can help you and your family https://www.familyservices.govt.nz/directory/

MDMA-assisted therapy in advanced-stage cancer (EMMAC study)

CONSENT FORM

1. I have read, or have had read to me in my first language, and I understand the Participant Information Sheet.
2. I have been given sufficient time to consider whether or not to participate in this study.
3. I have had the opportunity to use a legal representative, whānau/ family support or a friend to help me ask questions and understand the study.
4. I am satisfied with the answers I have been given regarding the study and I have a copy of this consent form and information sheet.
5. I understand that taking part in this study is voluntary (my choice) and that I may withdraw from the study at any time without this affecting my medical care.
6. I consent to the research staff collecting and processing my information, including information about my health.
7. If I decide to withdraw from the study, I agree that the information collected about me up to the point when I withdraw may continue to be processed.
8. I consent to my GP or current provider being informed about my participation in the study and of any significant abnormal results obtained during the study.
9. I understand that there may be risks associated with the treatment in the event of myself or my partner becoming pregnant. I undertake to inform my partner of the risks and to take responsibility for the prevention of pregnancy.
10. I understand that I am required to elect a support person who I will stay with after the dosing session and I have the option for them to be present for all sessions including therapy sessions.
11. I agree to an approved auditor appointed by the New Zealand Health and Disability Ethic Committees, or any relevant regulatory authority or their approved representative reviewing my relevant medical records for the sole purpose of checking the accuracy of the information recorded for the study.
12. I understand that my participation in this study is confidential and that no material which could identify me personally will be used in any reports on this study.
13. I understand the compensation provisions in case of injury during the study.
14. I know who to contact if I have any questions about the study in general.
15. I understand my responsibilities as a study participant.

16. I consent to having my visits to the research clinic video-recorded to be seen only by qualified therapists and supervisors for assessing the quality of therapy.

17. I wish to receive a summary of the results from the study. Yes ☐ No ☐

Declaration by participant:
I hereby consent to take part in this study.

Participant's name: ______________________________

Signature: ______________________ Date: ______________

Declaration by member of research team:

I have given a verbal explanation of the research project to the participant and have answered the participant's questions about it.

I believe that the participant understands the study and has given informed consent to participate.

Researcher's name: ______________________________

Signature: ______________________ Date: ______________
