# Supplementary material for: Effect of MDMA-assisted therapy on mood and anxiety symptoms in advanced-stage cancer (EMMAC): study protocol for a double-blind, randomised controlled trial
Source: Trials. 2024 May 21;25:336. doi: 10.1186/s13063-024-08174-x (PMC11110200; doi:10.1186/s13063-024-08174-x)
Supplement: Supplementary file 2 — Additional file 2. HDEC approval EMMAC. Ethics approval document from the Health and Disability Ethics Committee. [file 13063_2024_8174_MOESM2_ESM.pdf]

Health and Disability Ethics Committees

Health and Disability Ethics Committees
Ministry of Health
133 Molesworth Street
PO Box 5013
Wellington
6011
hdecs@health.govt.nz

Ethics reference: 21/NTB/105

22 September 2021

Professor Paul Glue

Department of Psychological Medicine ,University of Otago ,PO BOX 54
Dunedin
9056
New Zealand

Tēnā koe Professor Glue

APPROVAL OF APPLICATION

Study title: Effect of MDMA-assisted therapy on Mood and Anxiety Symptoms in Advanced-stage Cancer study: a Double-blind placebo-controlled pilot study

I am pleased to advise that your application was **approved** by the Northern B Health and Disability Ethics Committee (the Committee) on 22 September 2021. This decision was made through the full review pathway.

Conditions of HDEC approval

HDEC approval for this study is subject to the following conditions being met prior to the commencement of the study in New Zealand. It is your responsibility, and that of the study's sponsor, to ensure that these conditions are met. No further review by the Committee is required.

Standard conditions:

- Before the study commences at *any* locality in New Zealand, all relevant regulatory approvals must be obtained.
- Before the study commences at *any* locality in New Zealand, it must be registered in a clinical trials registry. This should be a registry approved by the World Health Organization (such as the Australia New Zealand Clinical Trials Registry, www.anzctr.org.au or https://clinicaltrials.gov/).
- Before the study commences at *each given* locality in New Zealand, it must be authorised by that locality in Ethics RM. Locality authorisation confirms that the locality is suitable for the safe and effective conduct of the study, and that local research governance issues have been addressed.

After HDEC review

Please refer to the *SOPs* for HDEC requirements relating to amendments and other post-approval processes.

Your next progress report is due by 22 September 2022.

As your study is an intervention study involving a new medicine, all progress reports **must** be accompanied by an annual safety report. While there is no prescribed format for annual safety reports, they must be no longer than two pages in length, written in lay language, and include a brief description and analysis of:

new and relevant findings that may have a significant impact on the safety of participants

- the safety profile of the new medicine and its implications for participants, taking into account all safety data as well as the results of any relevant non-clinical studies
- the implications of safety data to the risk-benefit ratio for the intervention study, and whether study documentation has been or will be updated
- any measures taken or proposed to minimise risks. (Where such a proposed measure would be a substantial amendment, it must be submitted for HDEC review in the normal way).

For the avoidance of doubt, Development Safety Update Reports may serve as annual safety reports to HDECs provided that they contain the information outlined above. These summaries should be accompanied by comment from the New Zealand coordinating investigator of the study.

Please refer to paragraphs 206 to 208 of the *SOPs* for further information.

Participant access to compensation

The Committee is satisfied that your study is not a clinical trial that is to be conducted principally for the benefit of the manufacturer or distributor of the medicine or item being trialled. Participants injured as a result of treatment received as part of your study may therefore be eligible for publicly-funded compensation through the Accident Compensation Corporation.

Further information and assistance

Please contact the HDECs Secretariat at hdecs@health.govt.nz or visit our website at www.ethics.health.govt.nz for more information.

Nāku noa, nā

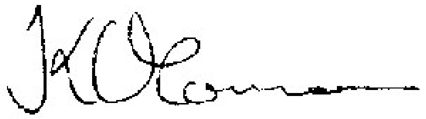

Ms Kate O'Connor

Chair

Northern B Health and Disability Ethics Committee

Encl: Appendix A: Documents submitted

Appendix B: Statement of compliance and list of members

Appendix A: Documents submitted

Document Type	File Name	Date	Version
MDF Doc	Form Submission	19/04/2021	NZ/1/53D919
Other	UoA HOD Approval letter	19/04/2021	Other (1)
Investigator's Brochure	13th Edition IB	19/04/2021	Investigator's Brochure (13)
CV for Coordinating Investigator	CV for Paul Glue	19/04/2021	CV for CI (11)
CV's for Other Investigators	CV Lisa Reynolds	19/04/2021	CVs for other Investigators (1)
CV's for Other Investigators	CV Will Evans	19/04/2021	CVs for other Investigators (1)
CV's for Other Investigators	CV David Menkes	19/04/2021	CVs for other Investigators (1)
CV's for Other Investigators	CV for Chris King	19/04/2021	CVs for other Investigators (1)
CV's for Other Investigators	CV for Nick Hoeh	19/04/2021	CVs for other Investigators (1)
CV's for Other Investigators	CV for Fred Sundrum	19/04/2021	CVs for other Investigators (1)
CV's for Other Investigators	CV Ingo Lambrecht	19/04/2021	CVs for other Investigators (1)
CV's for Other Investigators	CV for Jess Mills	19/04/2021	CVs for other Investigators (1)
CV's for Other Investigators	CV for Rhys Ponton	19/04/2021	CVs for other Investigators (1)
CV's for Other Investigators	CV for Shamsul Shah	19/04/2021	CVs for other Investigators (1)
CV's for Other Investigators	CV for Thivya Jeyaranjan	19/04/2021	CVs for other Investigators (1)
Evidence of Scientific Review	Debbie Bean review	19/04/2021	Evidence of scientific review (1)
Evidence of Scientific Review	Prof Ashton review	19/04/2021	Evidence of scientific review (1)
Other	Aotearoa/New Zealand Manual for MDMA-assisted psychotherapy treatment of depression and anxiety in the context of advanced-stage cancer	19/04/2021	Other (1)
PIS/CF	EMMAC Patient Information Sheet/Consent Form	19/04/2021	PIS/CF (1)
Other	Surveys/Questionnaires	19/04/2021	Survey/questionnaire (1)
Other	Hua Oranga Wellness scale	19/04/2021	Survey/questionnaire (1)
"Declined" letter for previous application	HDEC Letter Declined	19/04/2021	Declined letter for previous application in respect of the same (or substantially similar) study (1)
Other	MAPS Treatment Manual	19/04/2021	Other (1)
Covering Letter	Reply to HDEC letter	19/04/2021	Covering Letter (1)
Other	EMMAC reference manual	19/04/2021	Other (1)
Protocol	EMMAC Protocol	19/04/2021	Protocol (1)
Other	HDEC Letter_21NTB105_Valid Full Application.pdf	29/04/2021	HDEC Documents
Other	HDEC Letter_21NTB105_Full Application Provisionally Approved.pdf	13/05/2021	HDEC Documents
Other	Aotearoa/New Zealand Manual for MDMA-assisted psychotherapy treatment of depression and anxiety in the context of advanced-stage cancer	04/08/2021	Other (2)

PIS/CF	EMMAC Patient Information Sheet/Consent Form	04/08/2021	PIS/CF (2)
Protocol	EMMAC Protocol	04/08/2021	Protocol (1)
Covering Letter	Reply Cover letter	04/08/2021	Covering Letter (1)
Protocol	Updated Protocol	04/08/2021	Protocol (2)
Other	Updated Treatment Manual	04/08/2021	Other (2)
**Other**	Pamphlet for support person	04/08/2021	Other (1)
Other	Handover form template	04/08/2021	Other (1)
Other	ADHB restraint protocol	04/08/2021	Other (1)

Appendix B: Statement of compliance and list of members

Statement of compliance

The Northern B Health and Disability Ethics Committee

- is constituted in accordance with its Terms of Reference
- operates in accordance with the *Standard Operating Procedures for Health and Disability Ethics Committees*, and with the principles of international good clinical practice (GCP)
- is approved by the Health Research Council of New Zealand's Ethics Committee for the purposes of section 25(1)(c) of the Health Research Council Act 1990
- is registered (number 00008715) with the US Department of Health and Human Services' Office for Human Research Protection (OHRP).

List of members

Ms Kate O'Connor (lay ethical and moral reasoning), Mrs Leesa Russell (non-lay intervention/observational studies), Dr Gabrielle Jenkin (non-lay intervention/observational studies), Ms Susan Sherrard (lay consumer/community perspective), Mr Barry Taylor (non-lay intervention and observational studies), and Ms Maxine Shortland (lay consumer/community perspective).

Unless members resign, vacate or are removed from their office, every member of HDEC shall continue in office until their successor comes into office (HDEC Terms of Reference).

http://www.ethics.health.govt.nz
