# Supplementary material for: Effect of MDMA-assisted therapy on mood and anxiety symptoms in advanced-stage cancer (EMMAC): study protocol for a double-blind, randomised controlled trial
Source: Trials. 2024 May 21;25:336. doi: 10.1186/s13063-024-08174-x (PMC11110200; doi:10.1186/s13063-024-08174-x)
Supplement: Supplementary file 3 — Additional file 3. Funding Letter – EMMAC AUOA 2021. Funding documentation letter outlining funding received from an individual donor. [file 13063_2024_8174_MOESM3_ESM.pdf]

DocuSign Envelope ID: 7490D8A0-D280-47EF-8089-2CF906EAA06E

Saisei Foundation

July 12, 2021

Jonathan Wong, President
Alumni of University of Otago in America Inc.
495A Henry St #1040,
Brooklyn, NY 11231, United States

Dear Jonathan Wong:

As President of Saisei Foundation, I am pleased to inform you that the Foundation has approved a gift of $100,000.00 ("the Gift") to Alumni of University of Otago in America Inc., EIN # 30-0110891 ("the Grantee"), subject to the following conditions:

1. The Gift is for general support of the Grantee. With acknowledgement of the discretion of the Directors of the Grantee to make a grant to the University of Otago to benefit the University, the Foundation would like the Directors to note its preference that the gift be used to support the palliative care study with MAPS, University of Auckland and University of Otago led by Dr. William Evans.
2. The Foundation has made this grant in reliance on Grantee's IRS determination letter, the IRS Publication 78 Data that indicates Grantee is a public charity described in IRC Sections 501(c)(3) and 509(a)(1), and that Grantee's status has not changed. If at any time Grantee ceases to be so described, Grantee will return the unexpended portion of the Gift to the Foundation immediately. For so long as any amount of the Gift remains unexpended by Grantee, Grantee agrees promptly to notify the Foundation of a change or a proposed change to Grantee's tax-exempt status.

The Gift will be made by wire transfer within 10 days of this letter, provided that Grantee has confirmed to the Foundation its approval of its terms.

Yours truly,

DocuSigned by:
Tim Ferriss
E35F41B442E14C4...

Tim Ferriss
President

AGREED on behalf of Grantee:

[signature]

By: Jonathan Wong
Its: President

36703\14217295.4
